# Pan‐cancer landscape of tumour endothelial cells pinpoints insulin receptor as a novel antiangiogenic target and predicts immunotherapy response

**DOI:** 10.1002/ctm2.1501

**Published:** 2023-11-30

**Authors:** Yongqiang Zheng, Yi‐Qian Pan, Kun Liao, Kai Yu, Qinian Wu, Yanxing Chen, Yuqing Deng, Hui Sun, Hengying Pu, Huai‐Qiang Ju, Rui‐Hua Xu, Ze‐Xian Liu

**Affiliations:** ^1^ Department of Medical Oncology, State Key Laboratory of Oncology in South China, Collaborative Innovation Center for Cancer Medicine, Guangdong Key Laboratory of Nasopharyngeal Carcinoma Diagnosis and Therapy Sun Yat‐sen University Cancer Center Guangzhou P. R. China; ^2^ Research Unit of Precision Diagnosis and Treatment for Gastrointestinal Cancer Chinese Academy of Medical Sciences Guangzhou P. R. China

1

Dear Editor,

An in‐depth study of tumour vasculature can reveal the mechanisms and consequences of dysregulated tumour angiogenesis and influence new treatment strategies.^1^, ^2^, ^3^ Here, we establish the first pan‐cancer tumour endothelial cell (TEC) atlas, characterize the shared and cancer‐specific phenotypes of TECs among diverse cancer types and explore novel antiangiogenic targets.

Neo‐angiogenesis is an important step in tumour development and metastasis to meet the metabolic remodelling requirements of tumours.^4^ Anti‐angiogenic therapies have been devised to inhibit pathological angiogenesis, but their limited efficiency suggests that the urgency of more effective agents is needed.^5^ Deconstructing TECs at the single‐cell level could assist the development of new antiangiogenic drugs, but the current studies are limited by the narrowed cancer types, small sample sizes, and the relatively low abundance of endothelial cells (ECs) in tissue.^1^, ^2^, ^3^ Currently, there is a continuous lack of systematic studies to resolve TECs across different cancer types and in large sample sizes.

We established a pan‐cancer TEC atlas from the large‐scale data of a total of 1.24 million cells derived from 381 high‐quality samples (Figure S1A–G and Tables S1 and S2). A detailed description of this atlas is shown in the Supporting Information. Up to 63 320 ECs comprising 37 367 TECs and 25 953 normal ECs (NECs) were aggregated (Figure 1A–C). Clusters were annotated according to the biological functions of seven common EC lineages and the highest‐ranking marker genes in TECs and NECs,^3^ and 22 functional clusters were identified (Figure 1C–E). Hepatocellular carcinoma had a high arterial infiltration, and ovarian cancer had an angiogenic infiltration (Figures 1G and S2). Differentially expressed genes and pathways were used to validate the annotations (Figure S3).

**FIGURE 1:**
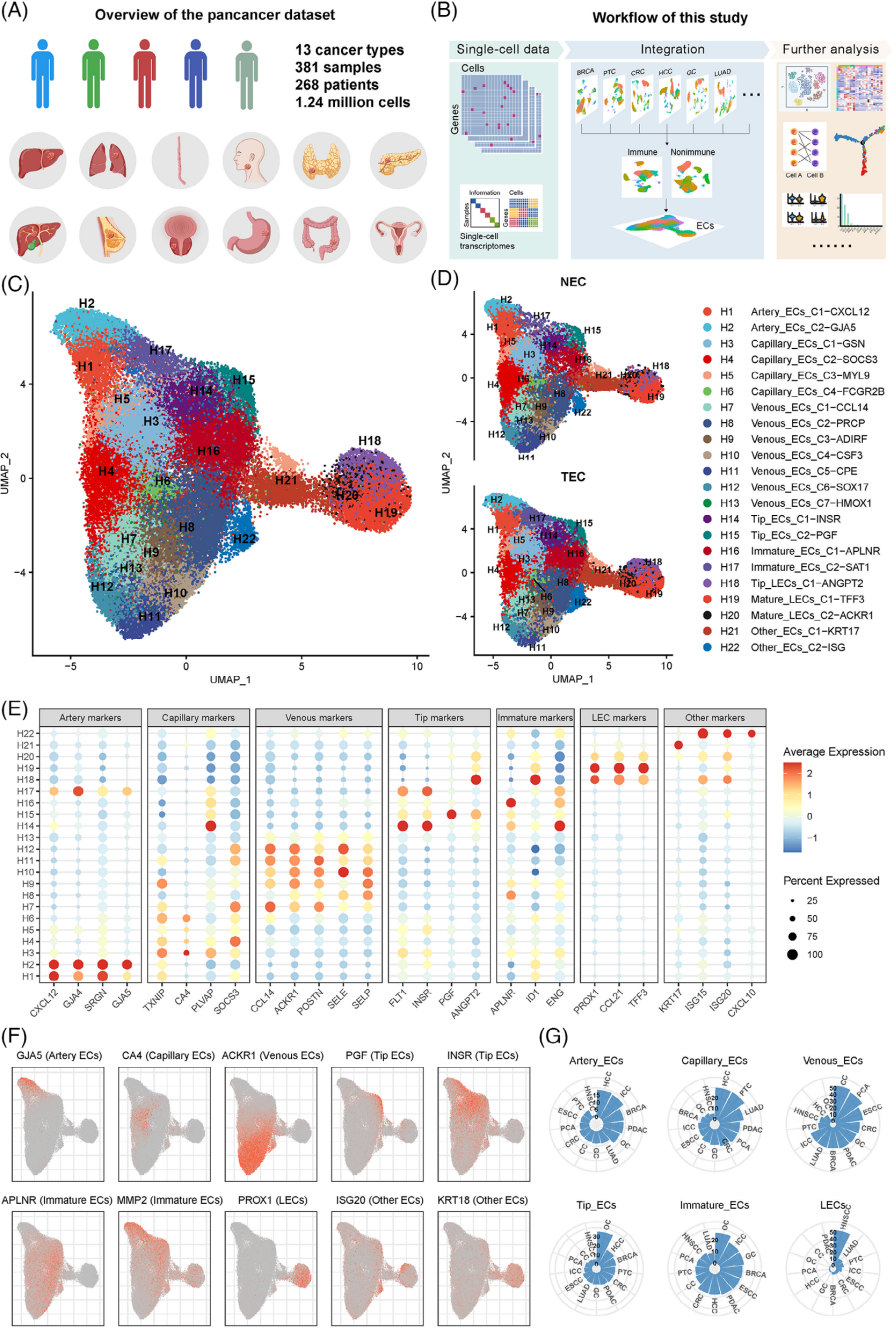
Pan‐cancer landscape of tumour endothelial cells (TECs) revealed by scRNA‐seq analysis. (A) Overview of the pan‐cancer datasets included in this study. (B) Workflow of this analysis process. (C) Uniform manifold approximation and projection (UMAP) plot of pan‐cancer scRNA‐seq EC data with 22 EC phenotypes. (D) UMAP plots of TECs and normal endothelial cells. (E) Dot plot of the gene expression levels of the top‐ranking marker genes in different EC phenotypes. (F) UMAP plots of the expression levels of the indicated EC marker genes. (G) Polar bar plot of the relative abundance of certain TEC phenotypes across diverse cancers. EC, endothelial cell; INSR, insulin receptor; LEC, lymphatic endothelial cells; NEC, normal EC.

This pan‐cancer TEC atlas revealed a profoundly altered construction of TECs, with markedly reduced functional components such as capillary ECs and significantly elevated angiogenic components, including tip and immature ECs (Figures 2A,B and S4A,B,I). Enrichment analyses demonstrated that TECs were enriched in pathways associated with angiogenesis, EC development, and EC proliferation (Figure 2C,D). Further investigations confirmed a consistent trend of decreasing capillary ECs and increasing angiogenic ECs across almost all cancers (Figure 2E–J). Scavenging FCGR2B+ capillary ECs were abundant in normal liver and lung^6^ but were substantially diminished in liver and lung tumours (Figure S4I–K). In addition, the proportion of tip lymphatic EC phenotypes was increased in tumours, indicating enhanced lymph‐angiogenesis in tumours (Figure 2G–J).

**FIGURE 2:**
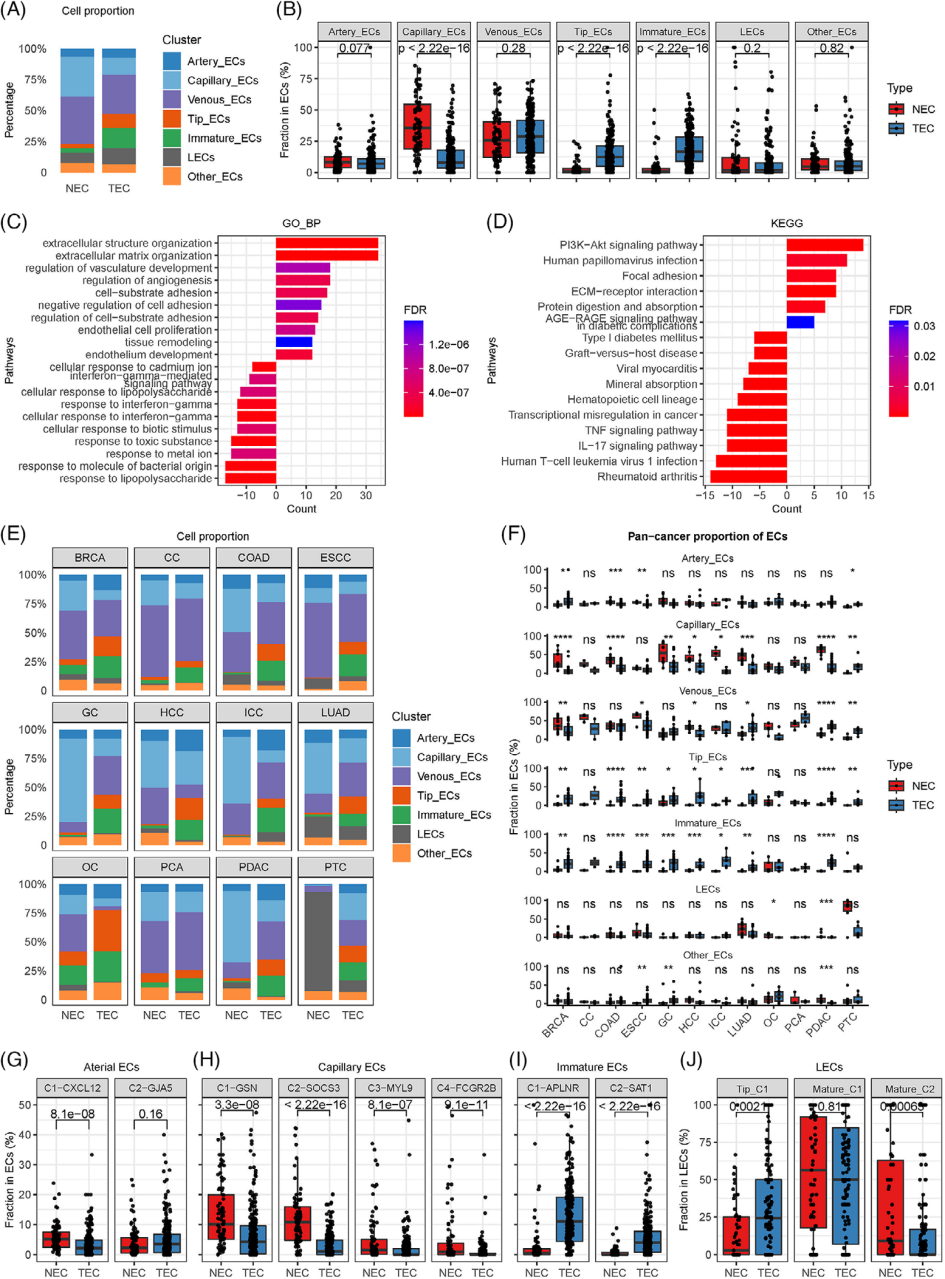
Comparisons of tumour endothelial cells (TECs) and normal endothelial cells (NECs) based on pan‐cancer single‐cell data. (A) Stacked bar plot comparing the relative abundance of major endothelial cell (EC) categories in TECs and NECs. (B) Box plot comparing the quantities of major EC categories of TECs and NECs (Wilcoxon test). The line and box represent the median and upper and lower quartiles, respectively. (C) Bar plot of the gene ontology biological process (GO BP) enrichment analyses of differentially expressed genes in TECs and NECs. (D) Bar plot of the Kyoto Encyclopedia of Genes and Genomes (KEGG) enrichment analyses of differentially expressed genes in TECs and NECs. (E) Stacked bar plot comparing the relative abundance of major EC categories in TECs and NECs in various cancer types. (F) Box plot comparing the quantities of major EC categories in TECs and NECs in various cancer types (Wilcoxon test). The line and box represent the median and upper and lower quartiles, respectively. *p < 0.05; **p < 0.05; ***p < 0.001; ****p < 0.0001; ns, not significant. (G) Box plot comparing the quantities of arterial EC phenotypes in TECs and NECs (Wilcoxon test). (H) Box plot comparing the quantities of capillary EC phenotypes in TECs and NECs (Wilcoxon test). (I) Box plot comparing the quantities of immature EC phenotypes in TECs and NECs (Wilcoxon test). (J) Box plot comparing the quantities of LEC phenotypes in tumour lymphatic endothelial cells (LECs) and normal LECs (Wilcoxon test). (G–J) The line and box represent the median and upper and lower quartiles, respectively. BRCA, breast cancer; CC, cervical cancer; ESCC, oesophageal squamous carcinoma; GC, gastric cancer; HCC, hepatocellular carcinoma; ICC, intrahepatic cholangiocarcinoma; LUAD, lung adenocarcinoma; PCA, prostate cancer; PDAC, pancreatic ductal adenocarcinoma.

Endothelial tip cells, the leading cells at the tips of vascular angiogenic sprouts, were universally increased in tumours (Figure 2); thus, comprehensive analyses of sprouting tip ECs were performed (Figure 3). Two novel tip EC clusters were newly defined, insulin receptor (INSR)+ tip ECs (C1) and PGF+ tip ECs (C2), which were verified in two independent cohorts (Figures 3A and S5). INSR+ tip ECs (C1), comprising the majority (67.6%) of all tip ECs, were significantly elevated in eight of the 12 cancer types (Figure 3B,C). The marker gene of this cluster, *INSR*, is a critical metabolic gene.^7^ INSR+ tip ECs expressed peaked levels of *VEGFR1* and *VEGFR2* (Figure 3D), suggesting their relation with angiogenesis.

**FIGURE 3:**
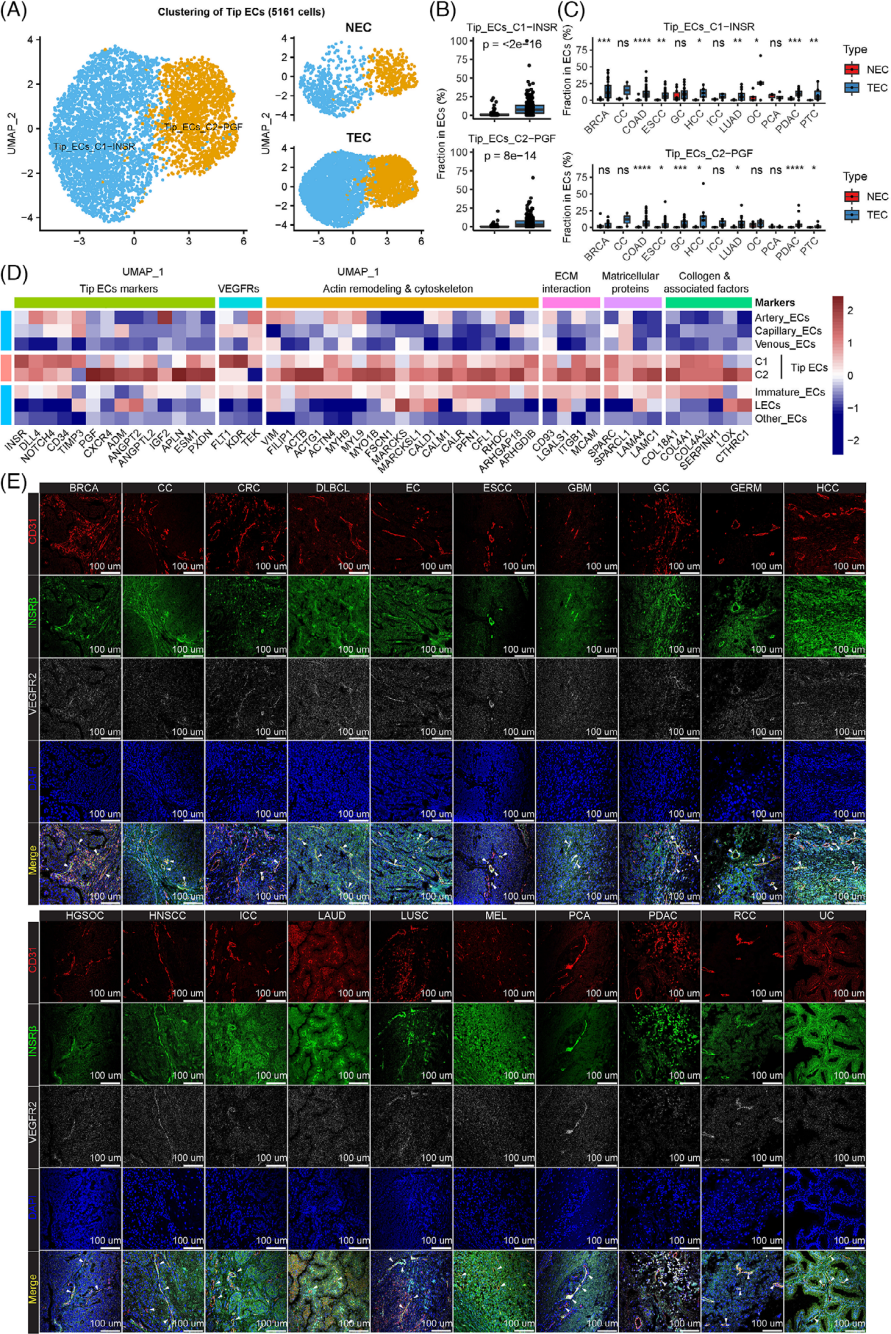
Clustering and validation of angiogenic tip endothelial cells (ECs). (A) Uniform manifold approximation and projection (UMAP) plot of tip tumour endothelial cells (TECs) and normal endothelial cells (NECs) (left: TECs and NECs combined; right: TECs and NECs separately). (B) Box plot comparing the quantities of INSR+ tip ECs and PGF+ tip ECs in TECs and NECs (Wilcoxon test). The line and box represent the median and upper and lower quartiles, respectively. (C) Box plot comparing the quantities of INSR+ tip ECs and PGF+ tip ECs in TECs and NECs in various cancer types (Wilcoxon test). The line and box represent the median and upper and lower quartiles, respectively. *p < 0.05; **p < 0.05; ***p < 0.001; ****p < 0.0001; ns, not significant. (D) Heatmap of gene expression levels of top‐ranking marker genes in the two tip EC phenotypes. (E) Multiplexed immunofluorescence (mIF) of CD31, INSRβ, VEGFR2 and DAPI in 20 types of cancer tissues. The following cancer types were included: breast cancer (BRCA), cervical cancer (CC), colorectal cancer (CRC), diffuse large B‐cell lymphoma (DLBCL), endometrial cancer (EC), oesophageal squamous carcinoma (ESCC), gastric cancer (GC), germ cell carcinoma (GERM), hepatocellular carcinoma (HCC), high‐grade serous ovarian cancer (HGSOC), intrahepatic cholangiocarcinoma (ICC), lung adenocarcinoma (LUAD), lung squamous carcinoma (LUSC), melanoma (MEL), prostate cancer (PCA), pancreatic ductal adenocarcinoma (PDAC), renal cell carcinoma (RCC) and urinary cancer (UC). INSR, insulin receptor; LECs, endothelial cells.

The expression of INSR in tumour vasculature was examined across diverse cancer types. Immunohistochemistry staining in 20 types of cancers exhibited a consistent amount of robust enrichment of INSR in TECs (Figure S6B,C). Further multiplex immunofluorescence staining of CD31, INSRβ, and VEGFR2 in 20 types of cancer tissues verified the uniform colocalization of INSRβ staining with CD31/VEGFR2 staining (Figure 3E). This universal pan‐cancer expression of INSR bolstered the credibility of INSR as a pan‐cancer angiogenic marker.

The enrichment analyses of INSR+ tip ECs yielded pathways related to angiogenesis and IGF binding that were upregulated (Figure 4A,B). In the IGF pathway, both *IGF1* and *IGF2* were significantly correlated with classical EC markers in multiple cancers, but the correlations between *IGF1R* or *IGF2R* and EC markers were weak (Figures 4C and S6A). INSR, another high‐affinity receptor of IGFs,^8^ was expressed exclusively in tumour ECs and identified as a novel angiogenic marker (Figure 4D,E). *INSR* strongly correlated with EC markers in multiple cancers (Figure S7A), indicating the pro‐angiogenic effects of IGFs via INSR. Further in vitro matrigel angiogenesis assays demonstrated that overexpression of *INSR* significantly enhanced the tube‐forming capacity (Figure 4F,H), while *INSR* knockdown impaired the formation of tubes (Figure 4G,I), supporting the enhancement of angiogenic capacity via INSR.

**FIGURE 4:**
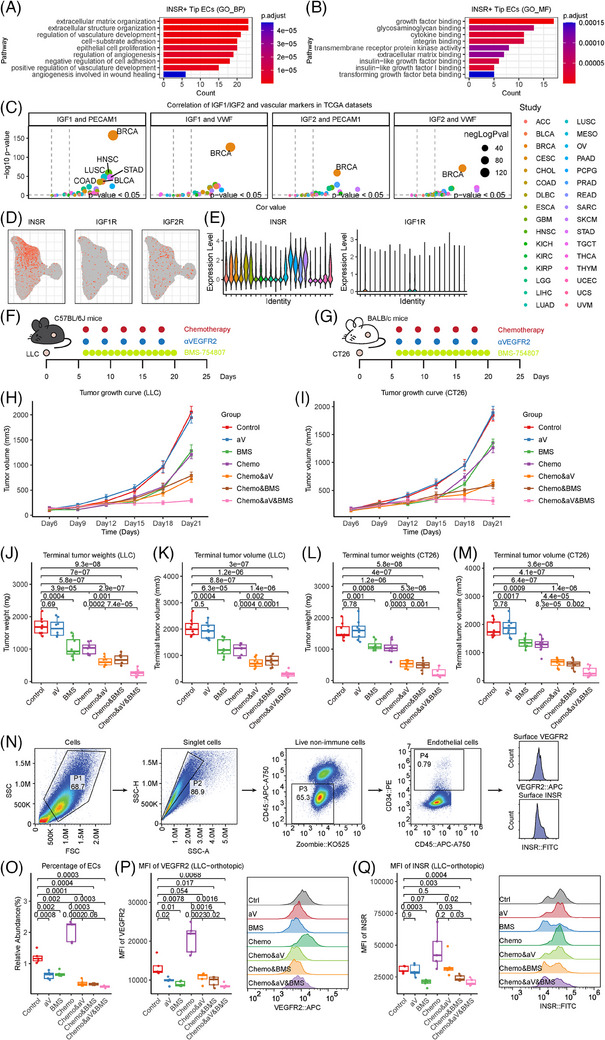
INSR is a promising novel antiangiogenic target. (A) Bar plot of the gene ontology biological process (GO BP) enrichment analyses of differentially expressed genes in INSR+ tip endothelial cells (ECs). (B) Bar plot of the gene ontology molecular function (GO MF) enrichment analyses of differentially expressed genes in INSR+ tip ECs. (C) Correlation of *IGF1* and *IGF2* with the common EC markers *CD31*/*PECAM1* and *VWF* in 32 cancer types in the TCGA database. The horizontal axis is the correlation coefficient, and the vertical axis is the negative logarithm of the *p* value. (D) Uniform manifold approximation and projection (UMAP) plot of ECs to show the expression of receptors of IGF, including *IGF1R*, *IGF2R* and *INSR*. (E) Violin plot of ECs showing the expression of IGF receptors, including *IGF1R* and *INSR*. (F–M) Subcutaneous tumour models were constructed to evaluate the treatment efficacy of αVEGFR2, BMS‐754807 or their combination with chemotherapy. (F,G) Scheme of the experiments. For LLC tumours (F), 1 × 10^6^ cells were subcutaneously injected into the flanks of C57BL/6J mice. Anti‐mouse VEGFR2 antibody and chemotherapy consisting of pemetrexed plus cisplatin were administered intraperitoneally every 3 days. BMS‐754807 was administered intraperitoneally daily. For CT26 tumours (G), 6 × 10^5^ cells were subcutaneously injected into the flanks of BALB/c mice. Anti‐mouse VEGFR2 antibody and chemotherapy consisting of pemetrexed plus cisplatin were administered intraperitoneally every 3 days. BMS‐754807 was administered intraperitoneally daily. (H,I) Growth curves of subcutaneous tumours derived from LLC or CT26 cells in different treatment groups. (J–M) Terminal tumour volumes and weights of subcutaneous tumours derived from LLC or CT26 cells in different treatment groups. The values are expressed as the mean ± standard error. (N,O) LLC orthotopic tumour models were constructed to evaluate the treatment efficacy of αVEGFR2, BMS‐754807 or their combination with chemotherapy. (N) Flow chart of flow cytometry to identify ECs and quantify the expression of INSR and VEGFR2 on ECs in LLC orthotopic models. (O) Boxplot showing the compositional changes of ECs by different interventions in LLC orthotopic models. (P) Boxplot (left) and rigid plot (right) showing the alteration of VEGFR2 expression in ECs by different interventions in LLC orthotopic models. (Q) Boxplot (left) and rigid plot (right) showing the alteration of INSR expression in ECs by different interventions in LLC orthotopic models. INSR, insulin receptor.

Then, we examined the in vivo therapeutic efficacy of BMS‐754807, a small‐molecule inhibitor of INSR,^9^ in subcutaneous tumour models of two mouse cell lines. Both LLC and CT26 tumours responded significantly to INSR inhibition, whether or not it was combined with chemotherapy. The efficacy reached maximum when chemotherapy, VEGFR2 antibody and INSR inhibitor were administered simultaneously (Figures 4J–Q and S7B,C).

We further used an orthotopic LLC model followed by flow cytometry to investigate the impacts of the INSR inhibitor on EC abundance (Figure 4N–Q). The INSR inhibitor significantly reduced the abundance of ECs in orthotopic tumours, regardless of whether chemotherapy was combined (Figures S7H and 4O). In addition, the INSR inhibitor could significantly lower the expression levels of VEGFR2 and INSR on ECs, and the effects were enhanced when αVEGFR2 was combined (Figure 4P,Q). Intriguingly, chemotherapy increased the EC abundance in orthotopic tumours, and the effects could be reversed by the INSR inhibitor (Figure 4O).

When comparing all EC clusters, we discovered that mature venous ECs expressed the highest level of MHC‐II molecules (Figure S8A,B). In two scRNA‐seq immunotherapy cohorts, we observed remarkably enriched MHC‐II+ mature venous ECs in patients with a better response to immunotherapy (Figure S8C–H). In addition, MHC‐II+ mature venous ECs were also linked with a significantly favourable prognosis after immunotherapy (Figure S8I–N).

In conclusion, we systematically characterized TECs across malignancies. Dissection of TECs revealed a novel INSR‐expressing tip TEC cluster as the predominant angiogenic driver. The pervasive presentation on angiogenic TECs implicates INSR as an angiogenic hallmark. The in vitro and in vivo experiments further verified INSR as a novel antiangiogenic target. Furthermore, MHC‐II+ mature venous ECs were linked with a better immunotherapy response. Our study of pan‐cancer TECs may facilitate the development of innovative antiangiogenic agents.

## AUTHOR CONTRIBUTIONS

Dr. Ze‐Xian Liu, Rui‐Hua Xu and Huai‐Qiang Ju had full access to all the data in the study and take responsibility for the integrity of the data and the accuracy of the data analysis. Dr. Yongqiang Zheng, Yi‐Qian Pan and Kun Liao contributed equally to this work as co‐first authors. Conception and design: Rui‐Hua Xu and Ze‐Xian Liu. Supervision: Rui‐Hua Xu and Ze‐Xian Liu. Acquisition, analysis, or interpretation of data: Yongqiang Zheng, Kai Yu, Yanxing Chen and Ze‐Xian Liu. Drafted the manuscript: Yongqiang Zheng, Yi‐Qian Pan, Yuqing Deng, Huai‐Qiang Ju, Ze‐Xian Liu and Rui‐Hua Xu. Experiments: Yi‐Qian Pan, Kun Liao, Yuqing Deng, Qinian Wu, Hui Sun and Huai‐Qiang Ju. Critical revision of the manuscript for important intellectual content: All authors. Software: Yongqiang Zheng, Kai Yu, Yanxing Chen and Hengying Pu. Statistical analysis: Yongqiang Zheng, Kai Yu and Ze‐Xian Liu. Obtained funding: Ze‐Xian Liu and Rui‐Hua Xu.

## CONFLICT OF INTEREST STATEMENT

The authors declare no conflicts of interest.

## ETHICAL STATEMENT

This study wasapproved by the Institutional Review Board of the Sun Yat‐sen University CancerCentre (No. B2023‐074‐01). The animal experiments in this study were approvedby the Animal Experiments Committee of Sun Yat‐sen University (No.L025501202303007).

## Supporting information

Supporting Information

Supporting Information
