# Supplementary material for: Pan‐cancer landscape of tumour endothelial cells pinpoints insulin receptor as a novel antiangiogenic target and predicts immunotherapy response
Source: Clin Transl Med. 2023 Nov 30;13(12):e1501. doi: 10.1002/ctm2.1501 (PMC10689971; doi:10.1002/ctm2.1501)
Supplement: Supplementary file 1 — Supporting Information [file CTM2-13-e1501-s002.docx]

**Supplementary Table 1** The information of data set included in the pan-cancer single cell analysis.

| **Cancer type** | **Cancer type (Full name)** | **Study** | **Patient number** | **Sample**  **number** | **Cell**  **number** | **Primary**  **Normal** | **Primary**  **Tumor** | **Lymph node metastasis** | **Brain**  **metastasis** | **PMID** |
| --- | --- | --- | --- | --- | --- | --- | --- | --- | --- | --- |
| BRCA | Breast cancer | BRCA_GSE161529 | 43 | 51 | 182,374 | Yes | Yes | Yes |  | 33950524 |
| CC | Cervical cancer | Cervical_E-MTAB-11948 | 3 | 6 | 46,454 | Yes | Yes |  |  | 36357663 |
| CRC | Colorectal cancer | CRC-SG1 | 14 | 67 | 176,482 | Yes | Yes |  |  | 32451460 |
| ESCC | Esophageal squamous cancer | ESCC_GSE160269 | 60 | 64 | 183,671 | Yes | Yes |  |  | 34489433 |
| GC | Gastric cancer | GC_GSE183904 | 27 | 34 | 107,508 | Yes | Yes |  |  | 34642171 |
| HCC | Hepatocellular carcinoma | HCC_GSE149614 | 10 | 18 | 56,695 | Yes | Yes | Yes |  | 35933472 |
| HNSCC | Head and neck squamous cell carcinoma | HNSCC_GSE164690 | 18 | 18 | 83,318 |  | Yes |  |  | 34921143 |
| ICC | Intrahepatic cholangiocarcinoma | ICC_GSE138709 | 5 | 8 | 25,226 | Yes | Yes |  |  | 32505533 |
| LUAD | Lung adenocarcinoma | LUAD_GSE131907 | 33 | 43 | 128,317 | Yes | Yes | Yes | Yes | 32385277 |
| OC | Ovarian cancer | OC_GSE184880 | 11 | 11 | 29,554 | Yes | Yes |  |  | 35675036 |
| PCA | Prostate cancer | PCA_GSE193337 | 1 | 7 | 11,443 | Yes | Yes |  |  | 35717322 |
| PDAC | Pancreatic ductal adenocarcinoma | PDAC_CRA001160 | 33 | 33 | 51,396 | Yes | Yes |  |  | 31273297 |
| PTC | Papillary thyroid carcinoma | PTC_GSE184362 | 10 | 21 | 158,588 | Yes | Yes | Yes |  | 34663816 |
| Total |  |  | 268 | 381 | 1,241,026 |  |  |  |  |  |

**Supplementary Table 2** The information of samples included in the pan-cancer single cell analysis.

| **Sample_ID** | **Study** | **Patient_ID** | **Sample.type** | **Gender** | **Age** | **TNM.stage** | **Stage** | **Differantiate** | **Type** | **Cancer.type** | **Cell_num** | **Note** |
| --- | --- | --- | --- | --- | --- | --- | --- | --- | --- | --- | --- | --- |
| BRCA_GSM4909253 | BRCA_GSE161529 | Patient_0092 | PN | F | 19 | PN | PN | NA | PN | BRCA | 4156 |  |
| BRCA_GSM4909254 | BRCA_GSE161529 | Patient_0019 | PN | F | 21 | PN | PN | NA | PN | BRCA | 5715 |  |
| BRCA_GSM4909257 | BRCA_GSE161529 | Patient_0093 | PN | F | 22 | PN | PN | NA | PN | BRCA | 3046 |  |
| BRCA_GSM4909261 | BRCA_GSE161529 | Patient_0230 | PN | F | 30 | PN | PN | NA | PN | BRCA | 3091 |  |
| BRCA_GSM4909263 | BRCA_GSE161529 | Patient_0064 | PN | F | 44 | PN | PN | NA | PN | BRCA | 1196 |  |
| BRCA_GSM4909265 | BRCA_GSE161529 | Patient_0233 | PN | F | 34 | PN | PN | NA | PN | BRCA | 4768 |  |
| BRCA_GSM4909266 | BRCA_GSE161529 | Patient_0169 | PN | F | 35 | PN | PN | NA | PN | BRCA | 3591 |  |
| BRCA_GSM4909268 | BRCA_GSE161529 | Patient_0123 | PN | F | 48 | PN | PN | NA | PN | BRCA | 5646 |  |
| BRCA_GSM4909270 | BRCA_GSE161529 | Patient_0342 | PN | F | 55 | PN | PN | NA | PN | BRCA | 7776 |  |
| BRCA_GSM4909271 | BRCA_GSE161529 | Patient_0288 | PN | F | 69 | PN | PN | NA | PN | BRCA | 1695 |  |
| BRCA_GSM4909272 | BRCA_GSE161529 | Patient_0021 | PN | F | 48 | PN | PN | NA | PN | BRCA | 1490 |  |
| BRCA_GSM4909274 | BRCA_GSE161529 | Patient_0275 | PN | F | 48 | PN | PN | NA | PN | BRCA | 1355 |  |
| BRCA_GSM4909276 | BRCA_GSE161529 | Patient_0372 | PN | F | 49 | PN | PN | NA | PN | BRCA | 2858 |  |
| BRCA_GSM4909281 | BRCA_GSE161529 | Patient_0126 | PT | F | 64 | NA | NA | NA | PT | BRCA | 2525 |  |
| BRCA_GSM4909282 | BRCA_GSE161529 | Patient_0135 | PT | F | 61 | NA | NA | NA | PT | BRCA | 13654 |  |
| BRCA_GSM4909283 | BRCA_GSE161529 | Patient_0106 | PT | F | 65 | NA | NA | NA | PT | BRCA | 816 |  |
| BRCA_GSM4909284 | BRCA_GSE161529 | Patient_0114 | PT | F | 84 | NA | NA | NA | PT | BRCA | 1393 |  |
| BRCA_GSM4909285 | BRCA_GSE161529 | Patient_4031 | PT | F | 25 | NA | NA | NA | PT | BRCA | 4918 |  |
| BRCA_GSM4909286 | BRCA_GSE161529 | Patient_0131 | PT | F | 84 | NA | NA | NA | PT | BRCA | 1197 |  |
| BRCA_GSM4909287 | BRCA_GSE161529 | Patient_0554 | PT | F | 29 | NA | NA | NA | PT | BRCA | 2433 |  |
| BRCA_GSM4909288 | BRCA_GSE161529 | Patient_0177 | PT | F | 30 | NA | NA | NA | PT | BRCA | 10734 |  |
| BRCA_GSM4909289 | BRCA_GSE161529 | Patient_0308 | PT | F | 32 | NA | NA | NA | PT | BRCA | 3117 |  |
| BRCA_GSM4909290 | BRCA_GSE161529 | Patient_0337 | PT | F | 66 | NA | NA | NA | PT | BRCA | 6738 |  |
| BRCA_GSM4909291 | BRCA_GSE161529 | Patient_0031 | PT | F | 47 | NA | NA | NA | PT | BRCA | 3525 |  |
| BRCA_GSM4909293 | BRCA_GSE161529 | Patient_0161 | PT | F | 80 | NA | NA | NA | PT | BRCA | 960 |  |
| BRCA_GSM4909294 | BRCA_GSE161529 | Patient_0176 | PT | F | 60 | NA | NA | NA | PT | BRCA | 5845 |  |
| BRCA_GSM4909295 | BRCA_GSE161529 | Patient_0319 | PT | F | 58 | NA | NA | NA | PT | BRCA | 2555 |  |
| BRCA_GSM4909296 | BRCA_GSE161529 | Patient_0001 | PT | F | 58 | NA | NA | NA | PT | BRCA | 4069 |  |
| BRCA_GSM4909297 | BRCA_GSE161529 | Patient_0125 | PT | F | 45 | NA | NA | NA | PT | BRCA | 3942 |  |
| BRCA_GSM4909298 | BRCA_GSE161529 | Patient_0360 | PT | F | 70 | NA | NA | NA | PT | BRCA | 2148 |  |
| BRCA_GSM4909299 | BRCA_GSE161529 | Patient_0114 | PT | F | 84 | NA | NA | NA | PT | BRCA | 7069 |  |
| BRCA_GSM4909300 | BRCA_GSE161529 | Patient_0032 | PT | F | 55 | NA | NA | NA | PT | BRCA | 915 |  |
| BRCA_GSM4909301 | BRCA_GSE161529 | Patient_0042 | PT | F | 58 | NA | NA | NA | PT | BRCA | 4158 |  |
| BRCA_GSM4909302 | BRCA_GSE161529 | Patient_0025 | PT | F | 52 | NA | NA | NA | PT | BRCA | 6873 |  |
| BRCA_GSM4909303 | BRCA_GSE161529 | Patient_0151 | PT | F | 49 | NA | NA | NA | PT | BRCA | 1100 |  |
| BRCA_GSM4909304 | BRCA_GSE161529 | Patient_0163 | PT | F | 45 | NA | NA | NA | PT | BRCA | 1233 |  |
| BRCA_GSM4909305 | BRCA_GSE161529 | Patient_0029 | PT | F | 59 | NA | NA | NA | PT | BRCA | 2447 |  |
| BRCA_GSM4909306 | BRCA_GSE161529 | Patient_0029 | PT | F | 59 | NA | NA | NA | PT | BRCA | 5670 |  |
| BRCA_GSM4909307 | BRCA_GSE161529 | Patient_0040 | PT | F | 69 | NA | NA | NA | PT | BRCA | 5320 |  |
| BRCA_GSM4909308 | BRCA_GSE161529 | Patient_0043 | mLN | F | 69 | NA | NA | NA | mLN | BRCA | 5195 |  |
| BRCA_GSM4909309 | BRCA_GSE161529 | Patient_0043 | PT | F | 55 | NA | NA | NA | PT | BRCA | 2620 |  |
| BRCA_GSM4909311 | BRCA_GSE161529 | Patient_0056 | PT | F | 66 | NA | NA | NA | PT | BRCA | 630 |  |
| BRCA_GSM4909312 | BRCA_GSE161529 | Patient_0056 | mLN | F | 66 | NA | NA | NA | mLN | BRCA | 5536 |  |
| BRCA_GSM4909313 | BRCA_GSE161529 | Patient_0064 | PT | F | 65 | NA | NA | NA | PT | BRCA | 2971 |  |
| BRCA_GSM4909314 | BRCA_GSE161529 | Patient_0064 | mLN | F | 65 | NA | NA | NA | mLN | BRCA | 707 |  |
| BRCA_GSM4909315 | BRCA_GSE161529 | Patient_0167 | PT | F | 83 | NA | NA | NA | PT | BRCA | 1902 |  |
| BRCA_GSM4909317 | BRCA_GSE161529 | Patient_0173 | PT | F | 83 | NA | NA | NA | PT | BRCA | 3428 |  |
| BRCA_GSM4909318 | BRCA_GSE161529 | Patient_0173 | mLN | F | 83 | NA | NA | NA | mLN | BRCA | 1560 |  |
| BRCA_GSM4909319 | BRCA_GSE161529 | Patient_0178 | PT | F | 84 | NA | NA | NA | PT | BRCA | 1563 |  |
| BRCA_GSM4909320 | BRCA_GSE161529 | Patient_0068 | PT | F | 79 | NA | NA | NA | PT | BRCA | 539 |  |
| BRCA_GSM4909321 | BRCA_GSE161529 | Patient_0068 | mLN | F | 79 | NA | NA | NA | mLN | BRCA | 3986 |  |
| GSM4909316 | Patient_0167 | BRCA_GSM4909316 | mLN | F | 83 | NA | NA | NA | mLN | BRCA | 411 | Discard |
| GSM4909292 | Patient_0069 | BRCA_GSM4909292 | PT | F | 71 | NA | NA | NA | PT | BRCA | 238 | Discard |
| Cervix_Sample1 | Cervical_E-MTAB-11948 | patient1 | PT | F | 48 | T1N0M0 | I | NA | PT | CC | 7952 |  |
| Cervix_Sample2 | Cervical_E-MTAB-11948 | patient2 | PT | F | 50 | T1N0M0 | I | NA | PT | CC | 5629 |  |
| Cervix_Sample3 | Cervical_E-MTAB-11948 | patient3 | PT | F | 51 | T1N0M0 | I | NA | PT | CC | 5920 |  |
| Cervix_Sample4 | Cervical_E-MTAB-11948 | patient1 | PN | F | 48 | PN | PN | NA | PN | CC | 11096 |  |
| Cervix_Sample5 | Cervical_E-MTAB-11948 | patient2 | PN | F | 50 | PN | PN | NA | PN | CC | 9988 |  |
| Cervix_Sample6 | Cervical_E-MTAB-11948 | patient3 | PN | F | 51 | PN | PN | NA | PN | CC | 5869 |  |
| CRC_MUX8563 | CRC-SG1 | CRC2794 | PT | F | 65 | T3N0M0 | II | NA | PT | COAD | 1338 |  |
| CRC_MUX8564 | CRC-SG1 | CRC2794 | PT | F | 65 | T3N0M0 | II | NA | PT | COAD | 1269 |  |
| CRC_MUX8565 | CRC-SG1 | CRC2794 | PT | F | 65 | T3N0M0 | II | NA | PT | COAD | 2561 |  |
| CRC_MUX8566 | CRC-SG1 | CRC2794 | PT | F | 65 | T3N0M0 | II | NA | PT | COAD | 2847 |  |
| CRC_MUX8567 | CRC-SG1 | CRC2794 | PN | F | 65 | T3N0M0 | II | NA | PN | COAD | 624 |  |
| CRC_MUX8568 | CRC-SG1 | CRC2795 | PT | M | 58 | T4N1M0 | III | NA | PT | COAD | 2138 |  |
| CRC_MUX8569 | CRC-SG1 | CRC2795 | PT | M | 58 | T4N1M0 | III | NA | PT | COAD | 1844 |  |
| CRC_MUX8570 | CRC-SG1 | CRC2795 | PT | M | 58 | T4N1M0 | III | NA | PT | COAD | 3035 |  |
| CRC_MUX8571 | CRC-SG1 | CRC2795 | PT | M | 58 | T4N1M0 | III | NA | PT | COAD | 1615 |  |
| CRC_MUX8572 | CRC-SG1 | CRC2795 | PN | M | 58 | T4N1M0 | III | NA | PN | COAD | 4861 |  |
| CRC_MUX8580 | CRC-SG1 | CRC2801 | PT | M | 50 | T4N2M0 | III | NA | PT | COAD | 511 |  |
| CRC_MUX8581 | CRC-SG1 | CRC2801 | PT | M | 50 | T4N2M0 | III | NA | PT | COAD | 2334 |  |
| CRC_MUX8582 | CRC-SG1 | CRC2801 | PT | M | 50 | T4N2M0 | III | NA | PT | COAD | 2512 |  |
| CRC_MUX8584 | CRC-SG1 | CRC2801 | PN | M | 50 | T4N2M0 | III | NA | PN | COAD | 3811 |  |
| CRC_MUX8631 | CRC-SG1 | CRC2803 | PT | M | 60 | T3N1M0 | III | NA | PT | COAD | 3209 |  |
| CRC_MUX8633 | CRC-SG1 | CRC2803 | PT | M | 60 | T3N1M0 | III | NA | PT | COAD | 2538 |  |
| CRC_MUX8639 | CRC-SG1 | CRC2803 | PT | M | 60 | T3N1M0 | III | NA | PT | COAD | 2108 |  |
| CRC_MUX8641 | CRC-SG1 | CRC2803 | PT | M | 60 | T3N1M0 | III | NA | PT | COAD | 1709 |  |
| CRC_MUX8643 | CRC-SG1 | CRC2803 | PN | M | 60 | T3N1M0 | III | NA | PN | COAD | 4188 |  |
| CRC_MUX8644 | CRC-SG1 | CRC2810 | PT | F | 76 | T4N2M0 | III | NA | PT | COAD | 1139 |  |
| CRC_MUX8645 | CRC-SG1 | CRC2810 | PT | F | 76 | T4N2M0 | III | NA | PT | COAD | 1737 |  |
| CRC_MUX8649 | CRC-SG1 | CRC2810 | PN | F | 76 | T4N2M0 | III | NA | PN | COAD | 2910 |  |
| CRC_MUX8722 | CRC-SG1 | CRC2811 | PT | M | 63 | T3N2M0 | III | NA | PT | COAD | 2152 |  |
| CRC_MUX8723 | CRC-SG1 | CRC2811 | PT | M | 63 | T3N2M0 | III | NA | PT | COAD | 2862 |  |
| CRC_MUX8724 | CRC-SG1 | CRC2811 | PT | M | 63 | T3N2M0 | III | NA | PT | COAD | 2472 |  |
| CRC_MUX8725 | CRC-SG1 | CRC2811 | PT | M | 63 | T3N2M0 | III | NA | PT | COAD | 2489 |  |
| CRC_MUX8726 | CRC-SG1 | CRC2811 | PN | M | 63 | T3N2M0 | III | NA | PN | COAD | 3462 |  |
| CRC_MUX8727 | CRC-SG1 | CRC2816 | PT | M | 59 | T3N1M0 | III | NA | PT | COAD | 3425 |  |
| CRC_MUX8728 | CRC-SG1 | CRC2816 | PT | M | 59 | T3N1M0 | III | NA | PT | COAD | 3503 |  |
| CRC_MUX8729 | CRC-SG1 | CRC2816 | PT | M | 59 | T3N1M0 | III | NA | PT | COAD | 4040 |  |
| CRC_MUX8730 | CRC-SG1 | CRC2816 | PT | M | 59 | T3N1M0 | III | NA | PT | COAD | 3571 |  |
| CRC_MUX8731 | CRC-SG1 | CRC2816 | PT | M | 59 | T3N1M0 | III | NA | PT | COAD | 5155 |  |
| CRC_MUX8732 | CRC-SG1 | CRC2816 | PN | M | 59 | T3N1M0 | III | NA | PN | COAD | 4282 |  |
| CRC_MUX8817 | CRC-SG1 | CRC2817 | PN | M | 67 | T4N1M0 | III | NA | PN | COAD | 3660 |  |
| CRC_MUX9005 | CRC-SG1 | CRC2817 | PT | M | 67 | T4N1M0 | III | NA | PT | COAD | 1644 |  |
| CRC_MUX9006 | CRC-SG1 | CRC2817 | PT | M | 67 | T4N1M0 | III | NA | PT | COAD | 3449 |  |
| CRC_MUX9007 | CRC-SG1 | CRC2817 | PT | M | 67 | T4N1M0 | III | NA | PT | COAD | 2714 |  |
| CRC_MUX9008 | CRC-SG1 | CRC2817 | PT | M | 67 | T4N1M0 | III | NA | PT | COAD | 1182 |  |
| CRC_MUX9009 | CRC-SG1 | CRC2817 | PT | M | 67 | T4N1M0 | III | NA | PT | COAD | 1166 |  |
| CRC_MUX9010 | CRC-SG1 | CRC2817 | PT | M | 67 | T4N1M0 | III | NA | PT | COAD | 610 |  |
| CRC_MUX9011 | CRC-SG1 | CRC2821 | PT | F | NA | T3N2M0 | III | NA | PT | COAD | 5666 |  |
| CRC_MUX9064 | CRC-SG1 | CRC2841 | PT | M | 64 | T4N2M0 | III | NA | PT | COAD | 3353 |  |
| CRC_MUX9065 | CRC-SG1 | CRC2841 | PT | M | 64 | T4N2M0 | III | NA | PT | COAD | 3232 |  |
| CRC_MUX9066 | CRC-SG1 | CRC2841 | PT | M | 64 | T4N2M0 | III | NA | PT | COAD | 1710 |  |
| CRC_MUX9067 | CRC-SG1 | CRC2841 | PN | M | 64 | T4N2M0 | III | NA | PN | COAD | 4912 |  |
| CRC_MUX9068 | CRC-SG1 | CRC2841 | PT | M | 64 | T4N2M0 | III | NA | PT | COAD | 2684 |  |
| CRC_MUX9069 | CRC-SG1 | CRC2841 | PT | M | 64 | T4N2M0 | III | NA | PT | COAD | 1477 |  |
| CRC_MUX9070 | CRC-SG1 | CRC2841 | PT | M | 64 | T4N2M0 | III | NA | PT | COAD | 1513 |  |
| CRC_MUX9071 | CRC-SG1 | CRC2841 | PT | M | 64 | T4N2M0 | III | NA | PT | COAD | 1285 |  |
| CRC_MUX9384 | CRC-SG1 | CRC2899 | PT | F | 76 | T3N1M0 | III | NA | PT | COAD | 3637 |  |
| CRC_MUX9385 | CRC-SG1 | CRC2899 | PT | F | 76 | T3N1M0 | III | NA | PT | COAD | 2186 |  |
| CRC_MUX9386 | CRC-SG1 | CRC2899 | PT | F | 76 | T3N1M0 | III | NA | PT | COAD | 3592 |  |
| CRC_MUX9387 | CRC-SG1 | CRC2899 | PT | F | 76 | T3N1M0 | III | NA | PT | COAD | 2646 |  |
| CRC_MUX9388 | CRC-SG1 | CRC2899 | PN | F | 76 | T3N1M0 | III | NA | PN | COAD | 5295 |  |
| CRC_XHC078 | CRC-SG1 | CRC2783 | PT | F | 31 | T3N1M0 | III | NA | PT | COAD | 1689 |  |
| CRC_XHC079 | CRC-SG1 | CRC2783 | PT | F | 31 | T3N1M0 | III | NA | PT | COAD | 990 |  |
| CRC_XHC080 | CRC-SG1 | CRC2783 | PT | F | 31 | T3N1M0 | III | NA | PT | COAD | 3973 |  |
| CRC_XHC081 | CRC-SG1 | CRC2783 | PT | F | 31 | T3N1M0 | III | NA | PT | COAD | 2792 |  |
| CRC_XHC082 | CRC-SG1 | CRC2783 | PT | F | 31 | T3N1M0 | III | NA | PT | COAD | 2368 |  |
| CRC_XHC083 | CRC-SG1 | CRC2783 | PN | F | 31 | T3N1M0 | III | NA | PN | COAD | 3620 |  |
| CRC_XHC084 | CRC-SG1 | CRC2787 | PT | F | 61 | T3N0M0 | II | NA | PT | COAD | 2360 |  |
| CRC_XHC085 | CRC-SG1 | CRC2787 | PT | F | 61 | T3N0M0 | II | NA | PT | COAD | 1622 |  |
| CRC_XHC087 | CRC-SG1 | CRC2786 | PT | M | 68 | T3N0M0 | II | NA | PT | COAD | 828 |  |
| CRC_XHC088 | CRC-SG1 | CRC2786 | PT | M | 68 | T3N0M0 | II | NA | PT | COAD | 655 |  |
| CRC_XHC089 | CRC-SG1 | CRC2786 | PT | M | 68 | T3N0M0 | II | NA | PT | COAD | 2059 |  |
| CRC_XHC090 | CRC-SG1 | CRC2786 | PT | M | 68 | T3N0M0 | II | NA | PT | COAD | 4052 |  |
| CRC_XHC091 | CRC-SG1 | CRC2786 | PN | M | 68 | T3N0M0 | II | NA | PN | COAD | 3610 |  |
| CRC16 | CRC2810 | CRC_MUX8648 | PT | F | 76 | T4N2M0 | III | NA | PT | CRC | 419 | Discard |
| CRC16 | CRC2801 | CRC_MUX8579 | PT | M | 50 | T4N2M0 | III | NA | PT | CRC | 392 | Discard |
| CRC16 | CRC2810 | CRC_MUX8647 | PT | F | 76 | T4N2M0 | III | NA | PT | CRC | 300 | Discard |
| CRC16 | CRC2817 | CRC_MUX9322 | PT | M | 67 | T4N1M0 | III | NA | PT | CRC | 120 | Discard |
| ESCC_P104T | ESCC_GSE160269 | P104 | PT | NA | NA | T3N2M0 | III | NA | PT | ESCC | 2256 |  |
| ESCC_P107T | ESCC_GSE160269 | P107 | PT | NA | NA | T1N0M0 | I | NA | PT | ESCC | 2528 |  |
| ESCC_P10T | ESCC_GSE160269 | P10 | PT | NA | NA | T3N0M0 | II | NA | PT | ESCC | 3820 |  |
| ESCC_P11T | ESCC_GSE160269 | P11 | PT | NA | NA | T3N0M0 | II | NA | PT | ESCC | 2759 |  |
| ESCC_P126N | ESCC_GSE160269 | P126 | PN | NA | NA | NA | PN | NA | PN | ESCC | 4545 |  |
| ESCC_P126T | ESCC_GSE160269 | P126 | PT | NA | NA | T3N1M0 | III | NA | PT | ESCC | 1434 |  |
| ESCC_P127N | ESCC_GSE160269 | P127 | PN | NA | NA | NA | PN | NA | PN | ESCC | 2357 |  |
| ESCC_P127T | ESCC_GSE160269 | P127 | PT | NA | NA | T3N2M0 | III | NA | PT | ESCC | 2474 |  |
| ESCC_P128N | ESCC_GSE160269 | P128 | PN | NA | NA | NA | PN | NA | PN | ESCC | 3273 |  |
| ESCC_P128T | ESCC_GSE160269 | P128 | PT | NA | NA | T2N1M0 | II | NA | PT | ESCC | 3095 |  |
| ESCC_P12T | ESCC_GSE160269 | P12 | PT | NA | NA | T3N0M0 | II | NA | PT | ESCC | 2343 |  |
| ESCC_P130N | ESCC_GSE160269 | P130 | PN | NA | NA | NA | PN | NA | PN | ESCC | 4608 |  |
| ESCC_P130T | ESCC_GSE160269 | P130 | PT | NA | NA | T3N0M0 | II | NA | PT | ESCC | 2045 |  |
| ESCC_P15T | ESCC_GSE160269 | P15 | PT | NA | NA | T3N0M0 | II | NA | PT | ESCC | 3301 |  |
| ESCC_P16T | ESCC_GSE160269 | P16 | PT | NA | NA | T2N0M0 | I | NA | PT | ESCC | 3222 |  |
| ESCC_P17T | ESCC_GSE160269 | P17 | PT | NA | NA | T2N0M0 | I | NA | PT | ESCC | 1801 |  |
| ESCC_P19T | ESCC_GSE160269 | P19 | PT | NA | NA | T3N0M0 | II | NA | PT | ESCC | 1729 |  |
| ESCC_P1T | ESCC_GSE160269 | P1 | PT | NA | NA | T1N0M0 | I | NA | PT | ESCC | 1464 |  |
| ESCC_P20T | ESCC_GSE160269 | P20 | PT | NA | NA | T3N2M0 | III | NA | PT | ESCC | 2353 |  |
| ESCC_P21T | ESCC_GSE160269 | P21 | PT | NA | NA | T3N2M0 | III | NA | PT | ESCC | 2944 |  |
| ESCC_P22T | ESCC_GSE160269 | P22 | PT | NA | NA | T3N0M0 | II | NA | PT | ESCC | 5388 |  |
| ESCC_P23T | ESCC_GSE160269 | P23 | PT | NA | NA | T3N3M0 | III | NA | PT | ESCC | 3182 |  |
| ESCC_P24T | ESCC_GSE160269 | P24 | PT | NA | NA | T3N1M0 | III | NA | PT | ESCC | 6152 |  |
| ESCC_P26T | ESCC_GSE160269 | P26 | PT | NA | NA | T3N2M0 | III | NA | PT | ESCC | 3205 |  |
| ESCC_P27T | ESCC_GSE160269 | P27 | PT | NA | NA | T3N0M0 | II | NA | PT | ESCC | 3596 |  |
| ESCC_P28T | ESCC_GSE160269 | P28 | PT | NA | NA | T3N1M0 | III | NA | PT | ESCC | 2694 |  |
| ESCC_P2T | ESCC_GSE160269 | P2 | PT | NA | NA | T1N0M0 | I | NA | PT | ESCC | 3342 |  |
| ESCC_P30T | ESCC_GSE160269 | P30 | PT | NA | NA | T1N0M0 | I | NA | PT | ESCC | 3040 |  |
| ESCC_P31T | ESCC_GSE160269 | P31 | PT | NA | NA | T2N0M0 | I | NA | PT | ESCC | 3494 |  |
| ESCC_P32T | ESCC_GSE160269 | P32 | PT | NA | NA | T3N0M0 | II | NA | PT | ESCC | 2822 |  |
| ESCC_P36T | ESCC_GSE160269 | P36 | PT | NA | NA | T3N0M0 | II | NA | PT | ESCC | 2466 |  |
| ESCC_P37T | ESCC_GSE160269 | P37 | PT | NA | NA | T3N1M0 | III | NA | PT | ESCC | 1471 |  |
| ESCC_P38T | ESCC_GSE160269 | P38 | PT | NA | NA | T3N0M0 | II | NA | PT | ESCC | 2786 |  |
| ESCC_P39T | ESCC_GSE160269 | P39 | PT | NA | NA | T2N1M0 | II | NA | PT | ESCC | 3761 |  |
| ESCC_P40T | ESCC_GSE160269 | P40 | PT | NA | NA | T2N2M0 | III | NA | PT | ESCC | 4230 |  |
| ESCC_P42T | ESCC_GSE160269 | P42 | PT | NA | NA | T1N1M0 | II | NA | PT | ESCC | 3475 |  |
| ESCC_P44T | ESCC_GSE160269 | P44 | PT | NA | NA | T3N2M0 | III | NA | PT | ESCC | 4365 |  |
| ESCC_P47T | ESCC_GSE160269 | P47 | PT | NA | NA | T3N2M0 | III | NA | PT | ESCC | 4117 |  |
| ESCC_P48T | ESCC_GSE160269 | P48 | PT | NA | NA | T1N0M0 | I | NA | PT | ESCC | 2939 |  |
| ESCC_P49T | ESCC_GSE160269 | P49 | PT | NA | NA | T2N0M0 | I | NA | PT | ESCC | 1515 |  |
| ESCC_P4T | ESCC_GSE160269 | P4 | PT | NA | NA | T2N0M0 | I | NA | PT | ESCC | 3585 |  |
| ESCC_P52T | ESCC_GSE160269 | P52 | PT | NA | NA | T2N0M0 | I | NA | PT | ESCC | 2584 |  |
| ESCC_P54T | ESCC_GSE160269 | P54 | PT | NA | NA | T3N0M0 | II | NA | PT | ESCC | 2946 |  |
| ESCC_P56T | ESCC_GSE160269 | P56 | PT | NA | NA | T1N0M0 | I | NA | PT | ESCC | 721 |  |
| ESCC_P57T | ESCC_GSE160269 | P57 | PT | NA | NA | T3N1M0 | III | NA | PT | ESCC | 2206 |  |
| ESCC_P5T | ESCC_GSE160269 | P5 | PT | NA | NA | T3N1M0 | III | NA | PT | ESCC | 4485 |  |
| ESCC_P61T | ESCC_GSE160269 | P61 | PT | NA | NA | T3N0M0 | II | NA | PT | ESCC | 2413 |  |
| ESCC_P62T | ESCC_GSE160269 | P62 | PT | NA | NA | T3N1M0 | III | NA | PT | ESCC | 2474 |  |
| ESCC_P63T | ESCC_GSE160269 | P63 | PT | NA | NA | T4N3M0 | III | NA | PT | ESCC | 1682 |  |
| ESCC_P65T | ESCC_GSE160269 | P65 | PT | NA | NA | T2N0M0 | I | NA | PT | ESCC | 3368 |  |
| ESCC_P74T | ESCC_GSE160269 | P74 | PT | NA | NA | T3N0M0 | II | NA | PT | ESCC | 1827 |  |
| ESCC_P75T | ESCC_GSE160269 | P75 | PT | NA | NA | T3N2M0 | III | NA | PT | ESCC | 2255 |  |
| ESCC_P76T | ESCC_GSE160269 | P76 | PT | NA | NA | T1N0M0 | I | NA | PT | ESCC | 3206 |  |
| ESCC_P79T | ESCC_GSE160269 | P79 | PT | NA | NA | T1N0M0 | I | NA | PT | ESCC | 2992 |  |
| ESCC_P80T | ESCC_GSE160269 | P80 | PT | NA | NA | T3N2M0 | III | NA | PT | ESCC | 2029 |  |
| ESCC_P82T | ESCC_GSE160269 | P82 | PT | NA | NA | T2N0M0 | I | NA | PT | ESCC | 995 |  |
| ESCC_P83T | ESCC_GSE160269 | P83 | PT | NA | NA | T3N3M0 | III | NA | PT | ESCC | 2402 |  |
| ESCC_P84T | ESCC_GSE160269 | P84 | PT | NA | NA | T3N1M0 | III | NA | PT | ESCC | 1994 |  |
| ESCC_P87T | ESCC_GSE160269 | P87 | PT | NA | NA | T3N2M0 | III | NA | PT | ESCC | 2581 |  |
| ESCC_P89T | ESCC_GSE160269 | P89 | PT | NA | NA | T3N1M0 | III | NA | PT | ESCC | 1234 |  |
| ESCC_P8T | ESCC_GSE160269 | P8 | PT | NA | NA | T4N1M0 | III | NA | PT | ESCC | 5004 |  |
| ESCC_P91T | ESCC_GSE160269 | P91 | PT | NA | NA | T3N0M0 | II | NA | PT | ESCC | 1788 |  |
| ESCC_P94T | ESCC_GSE160269 | P94 | PT | NA | NA | T3N1M0 | III | NA | PT | ESCC | 914 |  |
| ESCC_P9T | ESCC_GSE160269 | P9 | PT | NA | NA | T3N1M0 | III | NA | PT | ESCC | 5595 |  |
| GC_GSM5573466_sample1 | GC_GSE183904 | NGCII518 | PN | M | 74 | NA | PN | NA | PN | GC | 1621 |  |
| GC_GSM5573467_sample2 | GC_GSE183904 | NGCII518 | PT | M | 74 | NA | II | NA | PT | GC | 2089 |  |
| GC_GSM5573468_sample3 | GC_GSE183904 | NGCII519 | PT | M | 83 | NA | III | NA | PT | GC | 2867 |  |
| GC_GSM5573469_sample4 | GC_GSE183904 | NGCII520 | PN | M | 83 | NA | PN | NA | PN | GC | 1355 |  |
| GC_GSM5573470_sample5 | GC_GSE183904 | NGCII520 | PT | M | 83 | NA | III | NA | PT | GC | 2731 |  |
| GC_GSM5573471_sample6 | GC_GSE183904 | NGCII521 | PN | M | 62 | NA | PN | NA | PN | GC | 1400 |  |
| GC_GSM5573472_sample7 | GC_GSE183904 | NGCII521 | PT | M | 62 | NA | II | NA | PT | GC | 1289 |  |
| GC_GSM5573473_sample8 | GC_GSE183904 | NGCII524 | PT | M | 71 | NA | IV | NA | PT | GC | 6012 |  |
| GC_GSM5573474_sample9 | GC_GSE183904 | NGCII525 | PN | M | 73 | NA | PN | NA | PN | GC | 1910 |  |
| GC_GSM5573475_sample10 | GC_GSE183904 | NGCII525 | PT | M | 73 | NA | II | NA | PT | GC | 1918 |  |
| GC_GSM5573476_sample11 | GC_GSE183904 | NGCII527 | PN | M | 69 | NA | PN | NA | PN | GC | 742 |  |
| GC_GSM5573478_sample13 | GC_GSE183904 | NGCII529 | PT | F | 84 | NA | I | NA | PT | GC | 1692 |  |
| GC_GSM5573479_sample14 | GC_GSE183904 | NGCII502 | PT | M | 36 | NA | III | NA | PT | GC | 4952 |  |
| GC_GSM5573480_sample15 | GC_GSE183904 | NGCII511 | PT | M | 68 | NA | II | NA | PT | GC | 10475 |  |
| GC_GSM5573481_sample16 | GC_GSE183904 | NGCII499 | PT | M | 88 | NA | III | NA | PT | GC | 1353 |  |
| GC_GSM5573482_sample17 | GC_GSE183904 | NGCII509 | PT | M | 65 | NA | III | NA | PT | GC | 6752 |  |
| GC_GSM5573483_sample18 | GC_GSE183904 | NGCII498 | PT | F | 68 | NA | II | NA | PT | GC | 4712 |  |
| GC_GSM5573486_sample21 | GC_GSE183904 | NGCII513 | PN | M | 74 | NA | PN | NA | PN | GC | 1329 |  |
| GC_GSM5573488_sample23 | GC_GSE183904 | NGCII514 | PN | F | 67 | NA | PN | NA | PN | GC | 2095 |  |
| GC_GSM5573489_sample24 | GC_GSE183904 | NGCII514 | PT | F | 67 | NA | III | NA | PT | GC | 1706 |  |
| GC_GSM5573490_sample25 | GC_GSE183904 | NGCII512 | PN | F | 86 | NA | PN | NA | PN | GC | 3092 |  |
| GC_GSM5573491_sample26 | GC_GSE183904 | NGCII512 | PT | F | 86 | NA | III | NA | PT | GC | 2142 |  |
| GC_GSM5573492_sample27 | GC_GSE183904 | NGCII510 | PT | F | 66 | NA | I | NA | PT | GC | 3881 |  |
| GC_GSM5573493_sample28 | GC_GSE183904 | NGCII531 | PT | F | 70 | NA | III | NA | PT | GC | 4399 |  |
| GC_GSM5573494_sample29 | GC_GSE183904 | NGCII522 | PT | M | 64 | NA | II | NA | PT | GC | 2229 |  |
| GC_GSM5573495_sample30 | GC_GSE183904 | NGCII533 | PT | M | 55 | NA | III | NA | PT | GC | 3000 |  |
| GC_GSM5573496_sample31 | GC_GSE183904 | NGCII536 | PN | M | 63 | NA | PN | NA | PN | GC | 1222 |  |
| GC_GSM5573497_sample32 | GC_GSE183904 | NGCII539 | PT | F | 65 | NA | I | NA | PT | GC | 1291 |  |
| GC_GSM5573498_sample33 | GC_GSE183904 | NGCII540 | PT | M | 80 | NA | III | NA | PT | GC | 3691 |  |
| GC_GSM5573499_sample34 | GC_GSE183904 | NGCII541 | PT | M | 82 | NA | III | NA | PT | GC | 10136 |  |
| GC_GSM5573500_sample35 | GC_GSE183904 | NGCII538 | PN | F | 56 | NA | PN | NA | PN | GC | 3522 |  |
| GC_GSM5573501_sample36 | GC_GSE183904 | NGCII538 | PT | F | 56 | NA | IV | NA | PT | GC | 2768 |  |
| GC_GSM5573504_sample39 | GC_GSE183904 | NGCII543 | PT | F | 73 | NA | III | NA | PT | GC | 4012 |  |
| GC_GSM5573505_sample40 | GC_GSE183904 | NGCII545 | PT | M | 60 | NA | III | NA | PT | GC | 3123 |  |
| GSM5573477 | NGCII527 | GC_GSM5573477_sample12 | PT | M | 69 | NA | IV | NA | PT | GC | 489 | Discard |
| GSM5573487 | NGCII513 | GC_GSM5573487_sample22 | PT | M | 74 | NA | III | NA | PT | GC | 363 | Discard |
| HCC_HCC01T | HCC_GSE149614 | HCC01 | PT | Male | 66 | T1N0M0 | I | NA | PT | HCC | 2636 |  |
| HCC_HCC02T | HCC_GSE149614 | HCC02 | PT | Male | 65 | T1N0M0 | I | NA | PT | HCC | 3698 |  |
| HCC_HCC03N | HCC_GSE149614 | HCC03 | PN | Male | 66 | PN | I | NA | PN | HCC | 2066 |  |
| HCC_HCC03T | HCC_GSE149614 | HCC03 | PT | Male | 66 | T1N0M0 | I | NA | PT | HCC | 3747 |  |
| HCC_HCC04N | HCC_GSE149614 | HCC04 | PN | Male | 60 | PN | II | NA | PN | HCC | 2982 |  |
| HCC_HCC04T | HCC_GSE149614 | HCC04 | PT | Male | 60 | T2N0M0 | II | NA | PT | HCC | 2860 |  |
| HCC_HCC05N | HCC_GSE149614 | HCC05 | PN | Male | 65 | PN | III | NA | PN | HCC | 4283 |  |
| HCC_HCC05T | HCC_GSE149614 | HCC05 | PT | Male | 65 | T3N0M0 | III | NA | PT | HCC | 2527 |  |
| HCC_HCC06N | HCC_GSE149614 | HCC06 | PN | Female | 64 | PN | III | NA | PN | HCC | 3980 |  |
| HCC_HCC06T | HCC_GSE149614 | HCC06 | PT | Female | 64 | T3N0M0 | III | NA | PT | HCC | 3765 |  |
| HCC_HCC07N | HCC_GSE149614 | HCC07 | PN | Male | 48 | PN | III | NA | PN | HCC | 3391 |  |
| HCC_HCC08N | HCC_GSE149614 | HCC08 | PN | Male | 64 | PN | III | NA | PN | HCC | 4458 |  |
| HCC_HCC08T | HCC_GSE149614 | HCC08 | PT | Male | 64 | T4N0M0 | III | NA | PT | HCC | 4163 |  |
| HCC_HCC09N | HCC_GSE149614 | HCC09 | PT | Male | 48 | T4N0M0 | IV | NA | PT | HCC | 1712 |  |
| HCC_HCC09T | HCC_GSE149614 | HCC09 | PT | Male | 48 | T4N0M0 | IV | NA | PT | HCC | 2479 |  |
| HCC_HCC10L | HCC_GSE149614 | HCC10 | mLN | Male | 53 | T4N0M1 | IV | NA | mLN | HCC | 2528 |  |
| HCC_HCC10N | HCC_GSE149614 | HCC10 | PN | Male | 53 | PN | IV | NA | PN | HCC | 2879 |  |
| HCC_HCC10T | HCC_GSE149614 | HCC10 | PT | Male | 53 | T4N0M1 | IV | NA | PT | HCC | 2541 |  |
| HCC07T | HCC07 | HCC_HCC07T | PT | Male | 48 | T4N0M0 | III | NA | PT | HCC | 440 | Discard |
| HNSCC_HN01 | HNSCC_GSE164690 | HN01 | PT | M | 70-79 | T4N2M0 | IV | NA | PT | HNSCC | 6885 |  |
| HNSCC_HN02 | HNSCC_GSE164690 | HN02 | PT | F | 60-69 | T3N2M0 | III | NA | PT | HNSCC | 1048 |  |
| HNSCC_HN03 | HNSCC_GSE164690 | HN03 | PT | M | 80-89 | T4N0M0 | IV | NA | PT | HNSCC | 4983 |  |
| HNSCC_HN04 | HNSCC_GSE164690 | HN04 | PT | M | 50-59 | T3N1M0 | III | NA | PT | HNSCC | 1364 |  |
| HNSCC_HN05 | HNSCC_GSE164690 | HN05 | PT | F | 50-59 | T3N3M0 | III | NA | PT | HNSCC | 5190 |  |
| HNSCC_HN06 | HNSCC_GSE164690 | HN06 | PT | M | 30-39 | T3N0M0 | III | NA | PT | HNSCC | 2767 |  |
| HNSCC_HN07 | HNSCC_GSE164690 | HN07 | PT | M | 60-69 | T3N0M0 | III | NA | PT | HNSCC | 5003 |  |
| HNSCC_HN08 | HNSCC_GSE164690 | HN08 | PT | F | 70-79 | T1N0M0 | I | NA | PT | HNSCC | 2763 |  |
| HNSCC_HN09 | HNSCC_GSE164690 | HN09 | PT | F | 70-79 | T3N2M0 | III | NA | PT | HNSCC | 4618 |  |
| HNSCC_HN10 | HNSCC_GSE164690 | HN10 | PT | M | 50-59 | T3N0M0 | III | NA | PT | HNSCC | 4636 |  |
| HNSCC_HN11 | HNSCC_GSE164690 | HN11 | PT | M | 80-89 | T2N0M0 | II | NA | PT | HNSCC | 4991 |  |
| HNSCC_HN12 | HNSCC_GSE164690 | HN12 | PT | M | 50-59 | T2N1M0 | II | NA | PT | HNSCC | 5202 |  |
| HNSCC_HN13 | HNSCC_GSE164690 | HN13 | PT | M | 70-79 | T2N0M0 | II | NA | PT | HNSCC | 6785 |  |
| HNSCC_HN14 | HNSCC_GSE164690 | HN14 | PT | M | 50-59 | T1N1M0 | II | NA | PT | HNSCC | 4686 |  |
| HNSCC_HN15 | HNSCC_GSE164690 | HN15 | PT | F | 60-69 | T2N0M0 | II | NA | PT | HNSCC | 3633 |  |
| HNSCC_HN16 | HNSCC_GSE164690 | HN16 | PT | M | 40-49 | T2N1M0 | II | NA | PT | HNSCC | 5587 |  |
| HNSCC_HN17 | HNSCC_GSE164690 | HN17 | PT | M | 50-59 | T1N1M0 | II | NA | PT | HNSCC | 9308 |  |
| HNSCC_HN18 | HNSCC_GSE164690 | HN18 | PT | M | 50-59 | T2N2M0 | III | NA | PT | HNSCC | 3869 |  |
| ICC_GSM4116579_ICC_18_Adjacent | ICC_GSE138709 | ICC_18 | PN | F | NA | PN | PN | PN | PN | ICC | 8782 |  |
| ICC_GSM4116580_ICC_18_Tumor | ICC_GSE138709 | ICC_18 | PT | F | NA | T3N1Mx | III | Poorly | PT | ICC | 3322 |  |
| ICC_GSM4116581_ICC_20_Tumor | ICC_GSE138709 | ICC_20 | PT | F | NA | T2NxMx | II | Moderately | PT | ICC | 2122 |  |
| ICC_GSM4116582_ICC_23_Adjacent | ICC_GSE138709 | ICC_23 | PN | M | NA | PN | PN | PN | PN | ICC | 3872 |  |
| ICC_GSM4116583_ICC_23_Tumor | ICC_GSE138709 | ICC_23 | PT | M | NA | T2NxMx | II | Poorly | PT | ICC | 1489 |  |
| ICC_GSM4116584_ICC_24_Tumor1 | ICC_GSE138709 | ICC_24 | PT | M | NA | T2N0M0 | II | Moderately | PT | ICC | 2009 |  |
| ICC_GSM4116585_ICC_24_Tumor2 | ICC_GSE138709 | ICC_24 | PT | M | NA | T2N0M0 | II | Moderately | PT | ICC | 1739 |  |
| ICC_GSM4116586_ICC_25_Adjacent | ICC_GSE138709 | ICC_25 | PN | M | NA | PN | PN | PN | PN | ICC | 1891 |  |
| BRONCHO_11 | LUAD_GSE131907 | P1011 | mLN | NA | NA | NA | IV | NA | mLN | LUAD | 2845 |  |
| BRONCHO_58 | LUAD_GSE131907 | P1058 | PT | NA | NA | NA | IV | Poorly | PT | LUAD | 2519 |  |
| EBUS_06 | LUAD_GSE131907 | P1006 | PT | NA | NA | NA | IV | Poorly | PT | LUAD | 2042 |  |
| EBUS_10 | LUAD_GSE131907 | P1010 | mLN | NA | NA | NA | IV | NA | mLN | LUAD | 4580 |  |
| EBUS_12 | LUAD_GSE131907 | P1012 | mLN | NA | NA | NA | IV | NA | mLN | LUAD | 2732 |  |
| EBUS_13 | LUAD_GSE131907 | P1013 | mLN | NA | NA | NA | IV | Poorly | mLN | LUAD | 3205 |  |
| EBUS_15 | LUAD_GSE131907 | P1015 | mLN | NA | NA | NA | III | NA | mLN | LUAD | 943 |  |
| EBUS_19 | LUAD_GSE131907 | P1019 | mLN | NA | NA | NA | IV | NA | mLN | LUAD | 1765 |  |
| EBUS_28 | LUAD_GSE131907 | P1028 | PT | NA | NA | NA | IV | NA | PT | LUAD | 3789 |  |
| EBUS_49 | LUAD_GSE131907 | P1049 | PT | NA | NA | NA | IV | Poorly | PT | LUAD | 1575 |  |
| EBUS_51 | LUAD_GSE131907 | P1051 | mLN | NA | NA | NA | IV | NA | mLN | LUAD | 2900 |  |
| LUNG_N01 | LUAD_GSE131907 | P0001 | PN | NA | NA | NA | I | PN | PN | LUAD | 2834 |  |
| LUNG_N06 | LUAD_GSE131907 | P0006 | PN | NA | NA | NA | I | PN | PN | LUAD | 2576 |  |
| LUNG_N08 | LUAD_GSE131907 | P0008 | PN | NA | NA | NA | I | PN | PN | LUAD | 2987 |  |
| LUNG_N09 | LUAD_GSE131907 | P0009 | PN | NA | NA | NA | II | PN | PN | LUAD | 2274 |  |
| LUNG_N18 | LUAD_GSE131907 | P0018 | PN | NA | NA | NA | I | PN | PN | LUAD | 4108 |  |
| LUNG_N19 | LUAD_GSE131907 | P0019 | PN | NA | NA | NA | I | PN | PN | LUAD | 3499 |  |
| LUNG_N20 | LUAD_GSE131907 | P0020 | PN | NA | NA | NA | I | PN | PN | LUAD | 5119 |  |
| LUNG_N28 | LUAD_GSE131907 | P0028 | PN | NA | NA | NA | III | PN | PN | LUAD | 3011 |  |
| LUNG_N30 | LUAD_GSE131907 | P0030 | PN | NA | NA | NA | I | PN | PN | LUAD | 3468 |  |
| LUNG_N31 | LUAD_GSE131907 | P0031 | PN | NA | NA | NA | III | PN | PN | LUAD | 3775 |  |
| LUNG_N34 | LUAD_GSE131907 | P0034 | PN | NA | NA | NA | I | PN | PN | LUAD | 4584 |  |
| LUNG_T06 | LUAD_GSE131907 | P0006 | PT | NA | NA | NA | I | Moderately | PT | LUAD | 3084 |  |
| LUNG_T08 | LUAD_GSE131907 | P0008 | PT | NA | NA | NA | I | Moderately | PT | LUAD | 3407 |  |
| LUNG_T09 | LUAD_GSE131907 | P0009 | PT | NA | NA | NA | II | Poorly | PT | LUAD | 3472 |  |
| LUNG_T18 | LUAD_GSE131907 | P0018 | PT | NA | NA | NA | I | Moderately | PT | LUAD | 2781 |  |
| LUNG_T19 | LUAD_GSE131907 | P0019 | PT | NA | NA | NA | I | Well | PT | LUAD | 3933 |  |
| LUNG_T20 | LUAD_GSE131907 | P0020 | PT | NA | NA | NA | I | Poorly | PT | LUAD | 3625 |  |
| LUNG_T25 | LUAD_GSE131907 | P0025 | PT | NA | NA | NA | I | NA | PT | LUAD | 3928 |  |
| LUNG_T28 | LUAD_GSE131907 | P0028 | PT | NA | NA | NA | III | NA | PT | LUAD | 3864 |  |
| LUNG_T30 | LUAD_GSE131907 | P0030 | PT | NA | NA | NA | I | NA | PT | LUAD | 3695 |  |
| LUNG_T31 | LUAD_GSE131907 | P0031 | PT | NA | NA | NA | III | NA | PT | LUAD | 4860 |  |
| LUNG_T34 | LUAD_GSE131907 | P0034 | PT | NA | NA | NA | I | Moderately | PT | LUAD | 2453 |  |
| NS_02 | LUAD_GSE131907 | P3002 | mBrain | NA | NA | NA | IV | NA | mBrain | LUAD | 2011 |  |
| NS_03 | LUAD_GSE131907 | P3003 | mBrain | NA | NA | NA | IV | NA | mBrain | LUAD | 1749 |  |
| NS_04 | LUAD_GSE131907 | P3004 | mBrain | NA | NA | NA | IV | NA | mBrain | LUAD | 1449 |  |
| NS_06 | LUAD_GSE131907 | P3006 | mBrain | NA | NA | NA | IV | Poorly | mBrain | LUAD | 898 |  |
| NS_07 | LUAD_GSE131907 | P3007 | mBrain | NA | NA | NA | IV | NA | mBrain | LUAD | 4005 |  |
| NS_12 | LUAD_GSE131907 | P3012 | mBrain | NA | NA | NA | IV | NA | mBrain | LUAD | 2496 |  |
| NS_13 | LUAD_GSE131907 | P3013 | mBrain | NA | NA | NA | IV | NA | mBrain | LUAD | 3980 |  |
| NS_16 | LUAD_GSE131907 | P3016 | mBrain | NA | NA | NA | III | Poorly | mBrain | LUAD | 1113 |  |
| NS_17 | LUAD_GSE131907 | P3017 | mBrain | NA | NA | NA | IV | NA | mBrain | LUAD | 1706 |  |
| NS_19 | LUAD_GSE131907 | P3019 | mBrain | NA | NA | NA | IV | NA | mBrain | LUAD | 2678 |  |
| OC_GSM5599220_Norm1 | OC_GSE184880 | OC_Norm1 | PN | F | 55 | NA | PN | NA | PN | OC | 3063 |  |
| OC_GSM5599221_Norm2 | OC_GSE184880 | OC_Norm2 | PN | F | 47 | NA | PN | NA | PN | OC | 3574 |  |
| OC_GSM5599222_Norm3 | OC_GSE184880 | OC_Norm3 | PN | F | 46 | NA | PN | NA | PN | OC | 2417 |  |
| OC_GSM5599223_Norm4 | OC_GSE184880 | OC_Norm4 | PN | F | 51 | NA | PN | NA | PN | OC | 3798 |  |
| OC_GSM5599224_Norm5 | OC_GSE184880 | OC_Norm5 | PN | F | 49 | NA | PN | NA | PN | OC | 1455 |  |
| OC_GSM5599225_Cancer1 | OC_GSE184880 | OC_Cancer1 | PT | F | 50 | NA | III | NA | PT | OC | 2816 |  |
| OC_GSM5599226_Cancer2 | OC_GSE184880 | OC_Cancer2 | PT | F | 51 | NA | II | NA | PT | OC | 1302 |  |
| OC_GSM5599227_Cancer3 | OC_GSE184880 | OC_Cancer3 | PT | F | 41 | NA | I | NA | PT | OC | 2555 |  |
| OC_GSM5599229_Cancer5 | OC_GSE184880 | OC_Cancer5 | PT | F | 57 | NA | II | NA | PT | OC | 3162 |  |
| OC_GSM5599230_Cancer6 | OC_GSE184880 | OC_Cancer6 | PT | F | 48 | NA | III | NA | PT | OC | 2615 |  |
| OC_GSM5599231_Cancer7 | OC_GSE184880 | OC_Cancer7 | PT | F | 53 | NA | I | NA | PT | OC | 2797 |  |
| GSM5599228 | OC_Cancer4 | OC_GSM5599228_Cancer4 | PT | F | 47 | NA | I | NA | PT | OC | 416 | Discard |
| PCA_GSM5793824_P1n | PCA_GSE193337 | P1 | PN | M | 73 | PN | PN | NA | PN | PCA | 2011 |  |
| PCA_GSM5793825_P2n | PCA_GSE193337 | P2 | PN | M | 63 | PN | PN | NA | PN | PCA | 628 |  |
| PCA_GSM5793827_P4n | PCA_GSE193337 | P4 | PN | M | 72 | PN | PN | NA | PN | PCA | 2618 |  |
| PCA_GSM5793828_P1t | PCA_GSE193337 | P1 | PT | M | 73 | T2N0M0 | II | NA | PT | PCA | 1105 |  |
| PCA_GSM5793829_P2t | PCA_GSE193337 | P2 | PT | M | 63 | T2N0M0 | II | NA | PT | PCA | 1479 |  |
| PCA_GSM5793831_P3t | PCA_GSE193337 | P3 | PT | M | 61 | T3N0M0 | III | NA | PT | PCA | 1251 |  |
| PCA_GSM5793832_P4t | PCA_GSE193337 | P4 | PT | M | 72 | T3N0M0 | III | NA | PT | PCA | 2351 |  |
| GSM5793826 | P3 | PCA_GSM5793826_P3n | PN | M | 61 | PN | PN | NA | PN | PCA | 445 | Discard |
| PDAC_N1 | PDAC_CRA001160 | N1 | PN | F | 64 | NA | NA | normal pancreas | PN | PDAC | 2641 |  |
| PDAC_N10 | PDAC_CRA001160 | N10 | PN | F | 65 | NA | NA | normal pancreas | PN | PDAC | 1451 |  |
| PDAC_N11 | PDAC_CRA001160 | N11 | PN | F | 30 | NA | NA | normal pancreas | PN | PDAC | 1290 |  |
| PDAC_N2 | PDAC_CRA001160 | N2 | PN | M | 55 | NA | NA | normal pancreas | PN | PDAC | 1826 |  |
| PDAC_N4 | PDAC_CRA001160 | N4 | PN | M | 53 | NA | NA | normal pancreas | PN | PDAC | 918 |  |
| PDAC_N5 | PDAC_CRA001160 | N5 | PN | F | 52 | NA | NA | normal pancreas | PN | PDAC | 850 |  |
| PDAC_N6 | PDAC_CRA001160 | N6 | PN | F | 31 | NA | NA | normal pancreas | PN | PDAC | 678 |  |
| PDAC_N7 | PDAC_CRA001160 | N7 | PN | F | 42 | NA | NA | normal pancreas | PN | PDAC | 1063 |  |
| PDAC_N8 | PDAC_CRA001160 | N8 | PN | M | 41 | NA | NA | normal pancreas | PN | PDAC | 1107 |  |
| PDAC_N9 | PDAC_CRA001160 | N9 | PN | M | 34 | NA | NA | normal pancreas | PN | PDAC | 2303 |  |
| PDAC_T1 | PDAC_CRA001160 | T1 | PT | M | 64 | T4N2M0 | III | moderately-poorly differentiated | PT | PDAC | 998 |  |
| PDAC_T10 | PDAC_CRA001160 | T10 | PT | M | 61 | T2N1M0 | I | poorly differentiated | PT | PDAC | 760 |  |
| PDAC_T11 | PDAC_CRA001160 | T11 | PT | M | 51 | T3N1M0 | II | moderately-poorly differentiated | PT | PDAC | 2872 |  |
| PDAC_T12 | PDAC_CRA001160 | T12 | PT | M | 54 | T3N2M0 | III | poorly differentiated | PT | PDAC | 2058 |  |
| PDAC_T13 | PDAC_CRA001160 | T13 | PT | F | 58 | T2N1M0 | II | moderately-poorly differentiated | PT | PDAC | 1844 |  |
| PDAC_T14 | PDAC_CRA001160 | T14 | PT | F | 67 | T2N1M0 | II | well differentiated | PT | PDAC | 1900 |  |
| PDAC_T15 | PDAC_CRA001160 | T15 | PT | F | 54 | T2N1M0 | II | well differentiated | PT | PDAC | 1733 |  |
| PDAC_T16 | PDAC_CRA001160 | T16 | PT | F | 56 | T2N1M0 | II | poorly differentiated | PT | PDAC | 1448 |  |
| PDAC_T17 | PDAC_CRA001160 | T17 | PT | F | 71 | T2N0M0 | I | moderately differentiated | PT | PDAC | 1920 |  |
| PDAC_T18 | PDAC_CRA001160 | T18 | PT | F | 68 | T2N0M0 | I | moderately-poorly differentiated | PT | PDAC | 1410 |  |
| PDAC_T19 | PDAC_CRA001160 | T19 | PT | F | 59 | T2N0M0 | I | well-moderately differentiated | PT | PDAC | 2559 |  |
| PDAC_T2 | PDAC_CRA001160 | T2 | PT | M | 52 | T1N1M0 | II | well differentiated | PT | PDAC | 2688 |  |
| PDAC_T21 | PDAC_CRA001160 | T21 | PT | M | 59 | T2N0M0 | I | moderately-poorly differentiated | PT | PDAC | 740 |  |
| PDAC_T22 | PDAC_CRA001160 | T22 | PT | F | 67 | T2N0M0 | I | moderately differentiated | PT | PDAC | 2056 |  |
| PDAC_T23 | PDAC_CRA001160 | T23 | PT | M | 54 | T2N1M0 | II | moderately-poorly differentiated | PT | PDAC | 2504 |  |
| PDAC_T24 | PDAC_CRA001160 | T24 | PT | F | 44 | T1N0M0 | I | moderately differentiated | PT | PDAC | 1646 |  |
| PDAC_T3 | PDAC_CRA001160 | T3 | PT | F | 58 | T2N0M0 | I | moderately-poorly differentiated | PT | PDAC | 1218 |  |
| PDAC_T4 | PDAC_CRA001160 | T4 | PT | F | 72 | T1N1M0 | II | moderately differentiated | PT | PDAC | 914 |  |
| PDAC_T5 | PDAC_CRA001160 | T5 | PT | F | 65 | T2N0M0 | I | well-moderately differentiated | PT | PDAC | 938 |  |
| PDAC_T6 | PDAC_CRA001160 | T6 | PT | M | 64 | T3N0M0 | II | moderately-poorly differentiated | PT | PDAC | 1563 |  |
| PDAC_T7 | PDAC_CRA001160 | T7 | PT | M | 70 | T3N1M0 | II | moderately differentiated | PT | PDAC | 670 |  |
| PDAC_T8 | PDAC_CRA001160 | T8 | PT | F | 66 | T1N2M0 | III | moderately-poorly differentiated | PT | PDAC | 632 |  |
| PDAC_T9 | PDAC_CRA001160 | T9 | PT | M | 36 | T2N0M0 | II | moderately-poorly differentiated | PT | PDAC | 2198 |  |
| T20 | T20 | PDAC_T20 | PT | M | 59 | T3N1M0 | II | moderately differentiated | PT | PDAC | 459 | Discard |
| N3 | N3 | PDAC_N3 | PN | M | 50 | NA | NA | normal pancreas | PN | PDAC | 429 | Discard |
| PTC_GSM5585102_PTC1_T | PTC_GSE184362 | PTC1 | PT | NA | NA | T4N0M0 | IV | NA | PT | PTC | 5492 |  |
| PTC_GSM5585103_PTC1_P | PTC_GSE184362 | PTC1 | PN | NA | NA | PN | PN | NA | PN | PTC | 8380 |  |
| PTC_GSM5585104_PTC2_T | PTC_GSE184362 | PTC2 | PT | NA | NA | T4N1M0 | IV | NA | PT | PTC | 4818 |  |
| PTC_GSM5585105_PTC2_P | PTC_GSE184362 | PTC2 | PN | NA | NA | PN | PN | NA | PN | PTC | 7233 |  |
| PTC_GSM5585106_PTC2_LeftLN | PTC_GSE184362 | PTC2 | mLN | NA | NA | T4N1M0 | IV | NA | mLN | PTC | 9775 |  |
| PTC_GSM5585107_PTC3_T | PTC_GSE184362 | PTC3 | PT | NA | NA | T1N1M0 | IV | NA | PT | PTC | 6606 |  |
| PTC_GSM5585108_PTC3_P | PTC_GSE184362 | PTC3 | PN | NA | NA | PN | PN | NA | PN | PTC | 8617 |  |
| PTC_GSM5585109_PTC3_LeftLN | PTC_GSE184362 | PTC3 | mLN | NA | NA | T1N1M0 | III | NA | mLN | PTC | 9226 |  |
| PTC_GSM5585110_PTC3_RightLN | PTC_GSE184362 | PTC3 | mLN | NA | NA | T1N1M0 | III | NA | mLN | PTC | 8862 |  |
| PTC_GSM5585112_PTC5_T | PTC_GSE184362 | PTC5 | PT | NA | NA | T4N1M1 | IV | NA | PT | PTC | 3983 |  |
| PTC_GSM5585113_PTC5_P | PTC_GSE184362 | PTC5 | PN | NA | NA | PN | PN | NA | PN | PTC | 2453 |  |
| PTC_GSM5585114_PTC5_RightLN | PTC_GSE184362 | PTC5 | mLN | NA | NA | T4N1M1 | IV | NA | mLN | PTC | 5838 |  |
| PTC_GSM5585115_PTC6_RightLN | PTC_GSE184362 | PTC6 | mLN | NA | NA | rT0N1M0 | II | NA | mLN | PTC | 5362 |  |
| PTC_GSM5585116_PTC7_RightLN | PTC_GSE184362 | PTC7 | mLN | NA | NA | rT0N1M0 | II | NA | mLN | PTC | 7504 |  |
| PTC_GSM5585117_PTC8_T | PTC_GSE184362 | PTC8 | PT | NA | NA | T4N1M0 | IV | NA | PT | PTC | 8738 |  |
| PTC_GSM5585118_PTC8_P | PTC_GSE184362 | PTC8 | PN | NA | NA | PN | PN | NA | PN | PTC | 12892 |  |
| PTC_GSM5585119_PTC9_T | PTC_GSE184362 | PTC9 | PT | NA | NA | T1N1M0 | III | NA | PT | PTC | 9050 |  |
| PTC_GSM5585120_PTC9_P | PTC_GSE184362 | PTC9 | PN | NA | NA | PN | PN | NA | PN | PTC | 6391 |  |
| PTC_GSM5585121_PTC10_T | PTC_GSE184362 | PTC10 | PT | NA | NA | T4N1M1 | IV | NA | PT | PTC | 14509 |  |
| PTC_GSM5585122_PTC10_RightLN | PTC_GSE184362 | PTC10 | mLN | NA | NA | T4N1M1 | IV | NA | mLN | PTC | 6614 |  |
| PTC_GSM5585123_PTC11_RightLN | PTC_GSE184362 | PTC11 | mLN | NA | NA | rT0N1M1 | IV | NA | mLN | PTC | 6245 |  |
