# Supplementary material for: Pan‐cancer landscape of tumour endothelial cells pinpoints insulin receptor as a novel antiangiogenic target and predicts immunotherapy response
Source: Clin Transl Med. 2023 Nov 30;13(12):e1501. doi: 10.1002/ctm2.1501 (PMC10689971; doi:10.1002/ctm2.1501)
Supplement: Supplementary file 2 — Supporting Information [file CTM2-13-e1501-s001.docx]

**Supplementary Methods**

***Single-cell RNA-seq data integration and processing***

We aligned and quantified publicly available single-cell transcriptome data in FASTQ format published by 10x Genomics against the GRCh38 human reference genome using Cell Ranger software (Version 4.0.0) with default settings^1^. The Cell Ranger software's quantified count matrix and other publicly available count matrices were loaded into the Seurat tool (Version 4.1.1)^2^.

The cells were subjected to quality control based on many parameters. Cells with fewer than 500 identified genes and those with greater than 10% mitochondrial content were eliminated. To further exclude probable doublets, cells containing over 8000 identified genes were discarded. Possible doublets predicted by the DoubletFinder software were eliminated to avoid confounding the analysis^3^. Following filtering, samples containing fewer than 500 cells were deemed of poor quality and eliminated. Data from more than 1.24 million cells of good quality were retained for further analysis.

For the combined analysis of a large number of single-cell samples, the integration function was employed to combine all individual objects into a single aggregate object. We used the Seurat alignment workflow v4 to integrate all single-cell data in this study. The steps were: (1) We selected non-immune cells and immune cells from each dataset, and clustered non-immune cells and immune cells independently to reduce mutual interference. (2) Create a list of Seurat objects to integrate; (3) perform normalization, feature selection, and scaling separately for each dataset; (4) run principal component analysis on each object in the list; (5) integrate datasets, and proceed with joint analysis.

Using the FindVariableFeatures method, the top 3000 differentially expressed genes were returned for subsequent study. The dimensionality of this dataset was decreased using principal component analysis (PCA) with highly variable features, and the first 15 PCs were selected for investigation. To eliminate batch effects between datasets, the Harmony (Version 0.1.0) package was chosen for sample batch correction^4^. The FindNeighbors and FindClusters functions were used to provide unsupervised clustering based on the edge weights between any two cells and the shared nearest-neighbour graph generated by the Louvain algorithm. The detected clusters were displayed using the UMAP and TSNE approaches.

Data from each dataset were independently analysed using the aforementioned methods to identify immune cells and nonimmune cells. Immune cells and nonimmune cells from each sample were subsequently merged into two distinct datasets to prevent interaction. The same method was utilized for additional subclustering investigations, including normalization, variably expressed feature selection, dimension reduction, batch correction with Harmony, and clustering identification. To annotate the cell clusters, differentially expressed markers of the resulting clusters were discovered using the FindAllMarkers function and the Wilcoxon rank sum test with Bonferroni correction as the default nonparametric test.

***Cell–cell interaction analysis***

The CellChat program was utilized to study cell–cell interactions between ECs and TME components^5^. The files inputted into the statistical analysis function consisted of a raw count matrix and the relevant annotation file of cell types derived from the Seurat object. On the basis of their average expression, a visualization of the probable interaction intensity between ligand and receptor was projected. Significant ligand–receptor pairs were extracted for illustration.

***Single-cell trajectory analysis***

We used the Monocle (Version 2.18.0) algorithm to infer the interconversion and evolutionary paths to examine the plasticity and dynamic differentiation of ECs^6^. To reflect the intrinsic physiological properties of the cells, an unsupervised method was chosen. As the approach employed a raw count matrix of single-cell data, we used a single dataset to predict trajectory in an effort to reduce the impact of batch effects. Multiple individual datasets were analysed independently to verify the findings. The cells were reduced to two dimensions based on gene expression, and the orderCells function was then used to arrange them. Using differentialGeneTest and the "fullModelFormulaStr" option "sm.ns(Pseudotime)," pseudotime-dependent genes were identified, and smooth expression curves were created using the plot_pseudotime_heatmap function. Enrichment analyses based on the pseudotime-dependent genes in each state were then adopted to investigate the biological processes involved in cell differentiation.

***Bulk transcriptomic expression data***

Bulk transcriptomic expression counts and clinical information from the TCGA pan-cancer database were downloaded from XENA (http://xenabrowser.net/datapages/)^7^. Gene microarray and next-generation sequencing transcriptomic expression data were downloaded from the GEO database (https://www.ncbi.nlm.nih.gov/geo/) and ArrayExpress database (<https://www.ebi.ac.uk/biostudies/arrayexpress>)^8, 9^. For microarrays, the raw data were downloaded and then preprocessed using the oligo package to annotate the gene probe, perform background adjustments and remove batch effects.

***Enrichment analysis***

Differentially expressed markers of TECs and pseudotime-dependent genes from the Monocle algorithm were further subjected to GO and KEGG enrichment analysis using the clusterProfiler package (Version 3.0.4) with default settings^10^. The single-sample gene set variation analysis (ssGSVA) algorithm, a nonparametric and unsupervised algorithm from the gene set variation analysis (GSVA) package (Version 1.14.1)^11^, was selected to assess the TEC scores, tip EC scores and scores of other signatures such as hypoxia, in the single-cell dataset and bulk transcriptome datasets.

***Survival analysis***

The Survival (Version 2.42–3) and Survminer (Version 0.4.9) packages were used for analysis and visualization^12^. The best cut-off value for survival analysis inferred by the Survminer package was used to stratify the gene expression levels or the ssGSVA scores of a gene set.

***DNA construction***

Short hairpin RNA (shRNA) sequences targeting *INSR* were amplified by polymerase chain reaction (PCR) and cloned into psi-LVRU6P vector purchased from GeneCopoeia (Rockville, MD, USA). Sequence of *INSR* was amplified by PCR and cloned into pReceiver-Lv158 vector purchased from GeneCopoeia. The recombinant plasmids were sequenced to confirm the sequences were correct.

***Viral production and transfection***

Lentiviruses were produced by co-transfecting 2 μg of the constructed target plasmid with 2.8 μg packing plasmid psPAX2 and 1.56 μg envelope vector pMD2.G into HEK-293T cells cultured in T25 flasks using ViaFect Transfection Reagent (Promega, Madison, WI, USA) according to the manufacturer’s instructions. Lentiviruses were collected 72 hours after transfection. Virus transfection was performed by incubating cells with medium containing lentivirus and 8 μg/ml polybrene (OBio, Shanghai, China) for 24 hours. The efficiency of *INSR* knocking down or overexpression were validated by western blotting.

***Western blotting***

For western blotting, protein lysates were resolved by 5× loading buffer (Beyotime, Shanghai, China) and transferred to PVDF membranes with 0.45 μm pore (Millipore, MA, USA). All membranes were incubated with the indicated primary antibodies diluted in TBST (20 mM Tris pH 7.5, 150 mM NaCl, 0.1% Tween-20) supplemented with 5% bovine serum albumin (BSA, Sigma-Aldrich, MO, USA) overnight at 4℃. After 3 times of washing with TBST, membranes were incubated with horseradish peroxidase-conjugated secondary antibodies diluted in TBST-5%BSA for 1 hour at RT. Chemiluminescence was detected by ChemiDoc MP (Bio-Rad, CA, USA), following exposure SuperSignal West Femto Maximum Sensitivity Substrate (Thermo Fisher Scientific, MA, USA). Primary antibodies used in WB: anti-INSR-β (Cat#23413, Cell Signaling Technology, MA, USA), anti-α-Tubulin (Cat#2144, Cell Signaling Technology). Secondary antibody used in WB: goat anti-rabbit IgG, HRP-linked (Cat#7074, Cell Signaling Technology).

***IHC staining***

For IHC analysis, slides were baked in a dry oven for 2 hours at 65℃, then deparaffinized by incubation in 100% xylene for 15 min, twice, followed by rehydration (100% ethanol (EtOH), 95% EtOH, 70% EtOH, 100% dH2O for 5 min each). Endogenous peroxidase activity was blocked with 3% H2O2/Methanol solution for 10 min. Slides were then boiled in antigen unmasking solution (10 mM sodium citrate solution, pH 6.0) for 20 min, left to cool on bench for 20 min. Slides were then washed in PBS for 3×5 min and incubated in blocking buffer (5% BSA diluted in PBST buffer) for 1 hour at RT. Samples were incubated overnight with anti-INSR-β antibody (Cat#23413, Cell Signaling Technology, MA, USA) at a dilution of 1:200 at 4℃. Slides were washed in PBS for 3×5 min and then detected with Dako REAL EnVision Detection System, Peroxidase/DAB+. Rabbit/Mouse (Dako, Danmark). The stained slides were observed and photographed under Nikon Eclipse 80i Microscope (Nikon, Tokyo, Japan).

***mIF staining***

For mIF analysis, slides were baked in a dry oven for 2 hours at 65℃, then deparaffinized by incubation in 100% xylene for 15 min, twice, followed by rehydration (100% ethanol (EtOH), 95% EtOH, 70% EtOH, 100% dH2O for 5 min each). Slides were then boiled in antigen unmasking solution (10 mM sodium citrate solution, pH 6.0) for 20 min, left to cool on bench for 20 min. Slides were then washed in PBS for 3×5 min and incubated in blocking buffer (5% BSA diluted in PBST buffer) for 1 hour at RT. Samples were incubated overnight with anti-INSR-β antibody (Cat#23413, Cell Signaling Technology, MA, USA), anti-CD31 antibody (Cat#3528, Cell Signaling Technology) at a dilution of 1:500 at 4℃. Slides were washed in PBS for 3×5 min and then incubated with [Goat anti-Rabbit IgG (H+L) Cross-Adsorbed Secondary Antibody, Alexa Fluor^®^ 488](https://www.thermofisher.cn/antibody/product/Goat-anti-Rabbit-IgG-H-L-Cross-Adsorbed-Secondary-Antibody-Polyclonal/A-11008) (Cat#A-11008, ThermoFisher, MA, USA) [Goat anti-Mouse IgG (H+L) Cross-Adsorbed Secondary Antibody and Alexa Fluor^®^ 594](https://www.thermofisher.cn/antibody/product/Goat-anti-Mouse-IgG-H-L-Cross-Adsorbed-Secondary-Antibody-Polyclonal/A-11005) (Cat#A-11005, ThermoFisher) at a dilution of 1:1000 for 1 hour at RT. Slides were then washed in PBS for 3×5 min, incubated with Alexa Fluor^®^ 647 conjugated anti-VEGFR2 antibody (Cat#12658, Cell Signaling Technology) at a dilution of 1:200 for 1 hour at RT, washed in PBS for 3×5 min, and stained with DAPI. Immunofluorescence microscopic images of the cells were obtained using LSM880 and processed with ZEN software (Carl ZEISS, Jena, Germany).

***IHC, mIF and global expression of proteins in HPA database***

The HPA database provide IHC, mIF and global expression of proteins in both normal tissues and multiple tumour types^13^. We verified the markers used in single-cell clustering and annotation by the IHC, mIF images provided by HPA. Besides, the expression of individual genes and proteins in all types of tissues and cancers would aid us in determining the concentrated tissues and cells of certain proteins, and help us to identify ectopic gene expression in cancer.

***In vitro Matrigel angiogenesis assay***

Human Umbilical Vein Endothelial Cells (HUVECs) were cultured in Dulbecco’s Modified Eagle Medium (DMEM) with 10% fetal bovine serum and antibiotics, incubated in 5% CO2 at 37℃ in a humidified incubator. Before angiogenesis, HUVECs were serum starved for 24 h. Growth factor reduced Matrigel (Corning, NY, USA) were thawed at 4℃ and coated into 48-well plates with 150 μL of Matrigel per well. Let the plates sit at room temperature for 30 min to allow gelling of the Matrigel. HUVECs were digested with 0.25% trypsin, resuspended in DMEM and seeded onto the Matrigel at a density of 6×10^4 per well. After 3 h of tube formation at 37℃, cells were observed and photographed under Nikon ECLIPS Ti-2 microscope (Nikon, Tokyo, Japan).

***Animal experiment***

Six-week-old BALB/c or C57BL/6J mice were purchased from Beijing Vital River Laboratory Animal Technology Co., Ltd. LLC and CT26 cells were purchased form American Type Culture Collection (ATCC) and cultured under the recommended conditions. For LLC tumours, 1 × 10^6^ cells were subcutaneously injected into the flanks of C57BL/6J mice for the subcutaneous tumour model and 1 × 10^6^ cells were intrathoracically injected into the lungs of C57BL/6J mice for the orthotopic tumour model. Anti-mouse VEGFR2 antibody (Bioxcell, Cat#BE0060) and Chemotherapy consisting of pemetrexed (10 mg/kg) plus cisplatin (5 mg/kg) were administered intraperitoneally every three days. BMS-754807 (12.5 mg/kg) were dissolved in a solvent consisting of 10% DMSO, 30% PEG300, 5% Tween-80 and saline, and administered intraperitoneally daily. For CT26 tumours, 6 × 10^5^ cells were subcutaneously injected into the flanks of C57BL/6J mice. Anti-mouse VEGFR2 antibody (Bioxcell, Cat#BE0060) and Chemotherapy consisting of 5-fluorouracil (10 mg/kg) plus oxaliplatin (7.5 mg/kg) were administered intraperitoneally every three days. BMS-754807 (12.5 mg/kg, MCE, Cat# HY-10200) were administered intraperitoneally daily. Tumour size was routinely monitored every 3 days using a calliper. The experiment is terminated when the largest tumour exceeds 2 cm in long diameter or 2000 mm3 in volume. Tumours were dissected and photographed and terminal tumour weight were measured. Mice were euthanized at the end of experiments. This study has been approved by the Animal Experiments Committee of the Sun Yat-sen University (No. L025501202303007).

***Mouse tumour sample dissociation***

Mice were sacrificed for isolating tumour samples. Tumour samples were immediately minced into small pieces using a blade, which were then placed into 1 mL dissociating buffer configurated according to instruction of mouse tumour dissociation kit (Miltenyi, 130-096-730) at 37°C with rotation for 30 min. Then 70 μm and 40 μm cell strainers were applied in turns for sample filtration. MACS buffer was used for re-dissolving filtered cell mass and prepared for further staining.

***Flow cytometry***

Single cells prepared as described above were then stained with Zombie for 15 min at room temperature. PBS was used for washing. Then cells were stained with fluorophore-labelled antibodies against CD45 (Biolegend, Cat# 103154), CD31 (Biolegend, Cat# 102408), VEGFR2 (CST, Cat# 12658) and INSR (CST, Cat# 82011) for 20 min at room temperature. Cells were then washed and with fixation buffer (BD Biosciences, Cat# 554722) overnight at 4°C. Cells were evaluated by flow cytometry, and analysed with Flow Jo software.

***Immunotherapy cohort***

The roles of ECs in tumour immunobiology and immunotherapy response remain largely unknown, thus we then analysed the relationship between EC composition and immunotherapy response. PICC (ClinicalTrials.gov: NCT03926338) is a neoadjuvant platform trial investigating the combined effects of Toripalimab and celecoxib in mismatch repair-proficient and microsatellite-stable (dMMR/MSI-H) colorectal cancer (CRC)^14^. We extracted ECs from the scRNA-seq data of dMMR/MSI-H CRC in the PICC trial for further investigation. Major EC phenotypes were identified.

To increase the reliability, we extended this analysis to another independent cohort. TRACERx (ClinicalTrials.gov: NCT03226886) is a trial examining the efficiency of ICB (Nivolumab or Ipilimumab) on advanced clear cell renal cell carcinoma^15^.

Using ICB-treatment cohorts with transcriptome data obtained from the Tumor Immune Dysfunction and Exclusion database (TIDE), we estimated the level of MHC-II+ venous ECs using ssGSVA method and investigated its relationship with ICB response^16^. There ICB cohorts were included and analysed independently, including the Gide2019 melanoma cohort (Nivolumab or Pembrolizumab)^17^, Riaz2017 melanoma cohort (Nivolumab)^18^, and Prat2017 multiple cancer cohort (Pembrolizumab)^19^.

***Research Ethics***

This study has been approved by the Institutional Review Board of the Sun Yat-sen University Cancer Centre (No. B2023-074-01). The pan-cancer samples in mIF staining were retrospectively obtained from the preserved samples of Pathological Sample Bank in Sun Yat-sen University Cancer Centre. All the sample information has been anonymized. This study was performed according to the Declaration of Helsinki.

***General statistics***

When comparing data from different groups, t test, Wilcoxon test or one-way ANOVA was used (in R program version 4.0.3). Log-rank tests were adopted to test differences in survival. A 2-sided p value less than 0.05 was considered to indicate statistical significance.

***Data availability***

All the expression data can be obtained from the TCGA database, GEO database, or ArrayExpress database. The selected studies and related sample information are listed in **Supplementary Table 1**. Additionally, the integrated single-cell RNA sequencing matrix data that support the findings of this study are deposited publicly in the pan-cancer atlas of TECs database (<http://scrna.omicsbio.info>). Previously published scRNA-seq data that were reanalysed and integrated into this study are available under accession codes GSE161529 (BRCA), E-MTAB-11948 (cervical cancer), GSE132465 (CRC, CRC-SG1 dataset), GSE160269 (ESCC), GSE183904 (gastric cancer), GSE149614 (HCC), GSE164690 (HNSCC), GSE138709 (ICC), GSE131907 (LUAD), GSE184880 (ovarian cancer), CRA001160 (PDAC), GSE193337 (prostate cancer), and GSE184362 (PTC). Two single-cell transcriptome datasets of enriched ECs available under accession codes GSE155109 (BRCA CD31+ CD102+ cells) and E-MTAB-6308 (NSCLC CD31+ CD45- cells) were analysed independently as validation datasets. Processed publicly available transcriptomics data were obtained from the GEO database under accession codes GSE35894 (mouse hypoxia or normoxia CD31+ cells treated with or without L-685.458), GSE193424 (mouse LSECs with or without knock-out of the *RBPj* gene), GSE39413 (mouse glioblastoma xenograft model treated with or without dibenzazepine), GSE142322 (human stem cell-derived ECs treated with or without VEGFA for 8 h) and GSE23651 (rat cerebral model with or without ischaemic hypoxia). Our article is accompanied by an online data mining tool and a resource of pan-cancer single-cell EC atlas (<http://scrna.omicsbio.info>).

**Supplementary Interpretation of Results**

***Pan-cancer TME landscape illustrated through integrated scRNA-seq data***

To construct a comprehensive transcriptional atlas of TECs, we collected scRNA-seq data from 394 samples from 272 patients diagnosed with one of the 13 most prevalent types of cancers (**Figure 1A, Figure S1** and **Table S1**). Following rigorous quality control and filtering, we gathered a total of 1.24 million cells derived from the tumours, adjacent non-cancer tissues, or metastatic tumours (lymph node, brain) of 268 patients (381 high-quality samples) (**Figure 1A, Figure S1A-S1B** and **Table S1**). Except for head and neck squamous cell carcinoma (HNSCC), all the other 12 cancer types have both cancer and adjacent non-cancer tissues.

All eligible cells were subjected to a batch effect correction technique followed by unsupervised graph-based clustering. As seen in **Figure 1A** and **Figure S1A-S1B**, we selected non-immune cells and immune cells from each dataset, and clustered non-immune cells and immune cell independently to reduce mutual interference. We identify 14 common major lineages of immune cells, tumour cells, and stromal cells based on canonical cell markers, including B cells (*CD79A*, *MS4A1*), plasma cells (*MZB1*, *JCHAIN*), CD4+ Tconv cells (*CD3D*, *CD4*), CD4+ Treg cells (*FOXP3*), CD8+ T cells (*CD3D*, *CD8A*), mucosal-associated invariant T cells (*SLC4A10*, *KLRB1*), natural killer cells (*GNLY*, *NKG7*), dendritic cells (*CD1C*, MHC-II molecules), macrophages (*CD68*, *C1QC*), monocytes (*S100A9*, *CD14*), mast cells (*KIT*, *CPA3*), epithelial cells (*EPCAM*, *KRT8*), cancer-associated fibroblasts (CAFs: *LUM*, *COL1A1*), and ECs (*PECAM1*, *VWF*) (**Figure S1A**-S**1B**). Functional subclusters of these major lineages were identified and shown in **Figure 1C** and **Figure S1C-S1G**. Subsequently, ECs were annotated and selected out for further analyses. The underlying interrelationships may provide context for the application of AATs and immune checkpoint blockers in combination^20^. We investigated the expression levels of the *VEGF*, fibroblast growth factors, and insulin-like growth factor (*IGF*) families of growth factors and their receptors in the TMEs (**Figure S1H**). Multiple cell types in the TME including monocytes, epithelial cells, macrophages and CAFs release *VEGFA* in a dramatic manner. *PGF* is highly expressed in CAFs and ECs, whereas *ANGPT2* is found mostly in ECs. In addition, ECs express multiple vascular growth factor receptors including *FLT1*, *KDR* and *FLT4* which encodes VEGFR1, VEGFR2 and VEGFR3, respectively (**Figure S1H**)*.* Interestingly, *INSR*, which encodes insulin receptor, was also found to be upregulated exclusively in ECs (**Figure S1H**).

***Landscapes of TECs in 13 cancer types revealed by scRNA-seq analysis***

ECs were identified *in silico* (**Figures 1A**-**1B**), and up to 63,320 ECs comprising 37,367 TECs and 25,953 NECs were pooled as depicted by uniform manifold approximation and projection for dimension reduction (UMAP) plots (**Figure 1C**). Clusters were annotated according to biological functions and the highest-ranking marker genes in TECs and NECs, and finally 22 functional clusters were identified (**Figure 1C-1E**). The previously documented markers were used to infer the putative biological role of each cluster^21^. The distinctions in marker gene expression levels provided additional evidence for the qualitative biological annotation (**Figure 1C-1E**). Seven common EC lineages were identified: arterial ECs (*CXCL12*, *GJA5*), capillary ECs (*CA4*), venous ECs (*ACKR1*, *SELE*), tip ECs (*PGF*, *ESM1*, *CXCR4*), immature ECs (*APLNR*, *ENG*), lymphatic ECs (LECs: *PORX1*, *TFF3*) and other ECs (**Figure 1E-1F**). We analysed the abundance of each EC lineage in each tumour type and discovered that there was high heterogeneity in the EC components across cancer types (**Figure 1G**). We discovered that hepatocellular carcinoma (HCC) and intrahepatic cholangiocarcinoma (ICC) had a high number of arteries, which are exclusively nourished by the hepatic artery^22^, providing evidence to support treatments involving arterial occlusion^23^. Ovarian cancer possesses a greater proportion of tip ECs and immature ECs and has been demonstrated to be a suitable target for AATs^24^. HNSCC shows extensive LEC infiltration, explaining the frequent lymph node involvement and emphasizing the need for anti-LEC therapy^25^ (**Figure 1G**).

Vascular homeostasis, integrity, and vasoconstriction markers were substantially expressed in arterial ECs (clusters H1 and H2) (**Figure S2A**). A cluster expressing a high level of capillary EC markers (H3), an activated capillary EC cluster expressing EC activation markers (H4), a myoendothelial EC cluster expressing both EC markers and smooth muscle markers (H5), and a scavenging EC cluster expressing scavenging receptors and genes associated with macrophages (H6) were identified as functionally distinct clusters of capillary ECs (**Figure S2B**). Venous ECs (H7-H13) expressed genes involved in leukocyte recruitment, tissue perfusion, and antigen presentation mediated by MHC-II (**Figure S2C**). Venous ECs exhibited immune-related features. The presence of MHC-II molecules shows that venous ECs serve as antigen-presenting cells, and the cytokines by venous ECs can recruit immune cells (*CXCL1*, *CXCL8*, and *CSF3*). (**Figure S2C**). We identified three clusters of LECs: a tip LEC cluster (H18) expressing lymphangiogenesis-related and interferon-stimulated genes (ISGs) and two mature LEC clusters expressing primarily functional LEC markers (H19 and H20) (**Figure S2D**). The angiogenic characteristics of tip ECs (H14 and H15) are also visible in our scRNA-seq data (**Figure 1C**). In addition, we discovered two immature TEC phenotypes (H16 and H17) (**Figure 1C**) that resembled tip cells but expressed elevated levels of genes involved in the maturation of newly created vasculature and vascular barrier integrity, presumably suggesting stalk-like ECs (**Figure 1C**). In addition, we detected two distinct EC clusters, one resembled the epithelium and the other expressed ISG-related markers (H21 and H22). Kyoto Encyclopedia of Genes and Genomes (KEGG) enrichment analyses of differentially expressed genes verified the functions of the ECs such as vasoconstriction in arterial ECs (**Figure S2E**), complement and coagulation cascade in scavenging capillary ECs (**Figure S2F**), vascular smooth muscle contraction in myoendothelial ECs (**Figure S2F**), inflammatory and antigen-presentation status in venous ECs (**Figure S2G**), cytokine−cytokine receptor interaction in tip LECs, and proteoglycans in cancer and focal adhesion in mature LECs (**Figure S2H**).

***Characterization of TECs in contrast to NECs via pan-cancer analysis***

Compared to that of NECs, the construction of TECs was profoundly altered, with remarkably reduced functional components such as capillary ECs and significantly elevated angiogenic components including tip EC phenotypes and immature phenotypes (**Figure 2A**). The proportion of angiogenic tip ECs and immature ECs increased considerably in tumour samples when compared with normal samples (both p < 2.22e-16) (**Figure 2B**). Interestingly, increased angiogenic tip ECs and immature ECs could also be observed in metastatic tumour tissues (**Figure S3A-S3B** and **Figure S3I**).

Enrichment analyses on genes upregulated in TECs were further performed to elucidate the features of TECs. Gene Ontology (GO) enrichment analyses demonstrated that transcriptome profiles of TECs were enriched in pathways associated with vascular endothelium development, angiogenesis and EC proliferation, confirming the occurrence of robust angiogenesis in tumour TMEs, while pathways related to the inflammatory response and INFγ-related pathways were suppressed (**Figure 2C**). KEGG enrichment analyses suggested that the PI3K-Akt signalling pathway and focal adhesion pathway were activated in tumour tissues, whereas inflammatory pathways and TNF signalling pathways were downregulated in TECs (**Figure 2D**).

Further investigations on various cancer types revealed that, comparing the tumour-specific ECs and relevant NECs in the same organ, the fraction of angiogenic tip ECs and immature ECs was enhanced in nearly all cancer types (**Figure 2E**). While abundant in normal tissues of the colon, stomach, lung, liver, and pancreas, capillary endothelial cells were suppressed in the related tumour tissues. Venous ECs exhibited tumour-type specificity as significantly diminished in ovarian cancer but markedly increased in gastric cancer, PDAC, and papillary thyroid carcinoma (PTC). Arterial ECs were increased in BRCA, HCC, ICC, PDAC and PTC (**Figure 2E**). The normal thyroid gland was enriched in LECs, but the LECs in PTC were decreased. Further investigations confirmed the decrease in capillary ECs and the increase in tip ECs, and immature ECs as a consistent trend across almost all cancers (**Figure 2F**). The heterogeneity of the TECs across diverse cancer types illuminates the complexity and diversity of tumour vascularization in highly plastic tumour ecosystems, which hinders the application of AATs. Anti-tumour therapies require not only universal AATs but also individualized and specialized treatments due to the heterogeneity and uniqueness of vascularization across various cancer types.

Interestingly, PRCP+ venous ECs, which were previously reported to have an activated venous phenotype expressing *SELE*, *SELP* and *PRCP*^26^, were significantly increased in tumour tissues (**Figure 2G-2H**). CXCL12+ arterial ECs were significantly decreased in tumour tissues (**Figure S3C** and **Figure S3G**). Besides, the proportion of tip LEC phenotypes in all LECs was observed to be increased in tumours, presenting an increased lymphangiogenesis in tumours (**Figure S3F** and **Figure S3J**).

In malignancies, the significantly decreased capillary ECs (p < 2.22e-16), which is one of the major functional phenotypes, might reflect the disorganization of tumour vasculature (**Figure 2B, Figure S3D** and **Figure S3H**). The physically and functionally abnormal tumour vasculature disrupts tissue homeostasis, creating a pro-tumorigenic and immunosuppressive TME^27^. Besides, as capillary ECs are the major sites for the immune cells to entry tissues, the decrease in capillary ECs will form a physical barrier against cytotoxic immune cell infiltration. Besides, the capillary EC phenotypes with specific functions were also decreased in tumour tissues (**Figure S3D, Figure S3H and Figure S3K-S3M**). For example, FCGR2B+ capillary ECs with scavenging phenotype were abundant in normal liver and lung, but were significantly decreased in ICC, HCC and LUAD (**Figure S3K-S3M**). Scavenging ECs are the important subpopulation of liver sinusoids endothelial cells (LSECs) in the normal liver, and are specially equipped for the capacity to clean macromolecules and colloids by receptor-mediated endocytosis^28^. LSEC-derived scavenging ECs contribute importantly to maintain blood and tissue homeostasis by the removal of blood-borne wastes^28^. Unfortunately, the absence of scavenging capillary ECs in the cancerous liver leads to the disability of waste clearance (**Figure S3K-S3M**).

Various types of tumours exhibited features of disorganized tumour vasculature and enhanced angiogenesis/lymphangiogenesis, with reduced functional capillary ECs and consistently increased numbers of cells with angiogenic tip and immature EC phenotypes.

***Characterization of angiogenic tip ECs via pan-cancer analysis***

Tip ECs are the leading cells at the tips of vascular sprouts coordinating angiogenic processes. Sprouting angiogenesis requires hierarchical organization and activation of ECs from pre-existing blood vessels to form new vessels, while at the same time the majority of ECs need to remain quiescent. This hierarchical organization is mainly mediated by specialized motile ECs located at the tips of growing vessels, termed tip ECs. As they are the most important cells in angiogenic process, tip cells have a unique functional and molecular signature and are therefore an attractive target for therapeutic modulation of angiogenesis. In tumours, tip ECs were universal increasing (Figure 2), yielding enhanced angiogenesis. Therefore, we performed comprehensive analyses of sprouting tip ECs to investigate novel anti-angiogenic targets.

Given the universal increase of tip ECs in pan-cancer tissues, a detailed analysis of tip ECs was performed (**Figure 3**). Two clusters with tip EC phenotypes were newly defined after clustering and annotation: INSR+ tip ECs (C1) and PGF+ tip ECs (C2) (**Figure 3A**). INSR+ tip ECs were elevated in nearly all cancer types, with statistically significant increases in eight of the twelve cancer types. Similarly, PGF+ tip ECs were also significantly increased in multiple cancer types (**Figure 3B-3C**).

INSR+ tip ECs (C1), with an exclusively high expression of insulin receptor, are the major component of tip TECs, accounting for 67.6% of all tip ECs*.* Insulin receptor belongs to the receptor tyrosine kinase superfamily and plays a critical role in the regulation of human metabolism^29^. Previous study also reported a high expression of INSR in TECs^30, 31^, our study confirmed this finding and proposed it to be a new marker of tip ECs. Further analyses suggested that INSR+ tip ECs expressed peaked levels of *FLT1*/*VEGFR1* and *KDR*/*VEGFR2* (**Figure 3D** and **Figure S4A-S4B**), which suggested that this newly identified tip phenotype plays an important role in tumour angiogenesis. Besides, the high level of *CD34*, a marker of hematopoietic stem cells, indicated an elevated stemness in INSR+ tip ECs. The expression of stemness markers demonstrated its capacity of self-renewing and differentiation, which exhibits as an angiogenic phenotype. The other group of tip ECs was PGF+ tip ECs (C2), which expressed high levels of pro-angiogenic factors, including *PGF*, *ANGPT2*, *ANGPTL2* and *APLN* (**Figure 3D** and **Figure S4A-S4B**). *PGF*, which encodes placental growth factor (PLGF), is a key molecule in angiogenesis as a less common member of the VEGF family and binds to VEGFR1^32^. According to the gene expression data of all human tissue cell types from HPA databases, we found that PGF was expressed exclusively in trophoblast cells but rarely expressed in other tissues (**Figure S5A**). The abnormally high level of *PGF* in tumour tip ECs is an ideal example of ectopic gene expression in tumours, which might act as tumour-promoting factors.

In summary, two tip EC clusters with distinct phenotypes were newly identified: INSR+ tip ECs (C1) with an angiogenic phenotype and PGF+ tip ECs (C2) with a pro-angiogenic phenotype. To validate the characterization of these two tip EC phenotypes, two external scRNA-seq datasets with enriched TECs were analysed independently (**Figure S4C-S4H**). In the first TEC dataset of non-small cell lung cancer (NSCLC) from Goveia J. *et al*^26^, we identified these two clusters of tip ECs in scRNA-seq data of CD31+CD45- cells (**Figure S4C**). Both INSR+ tip ECs (p = 0.015) and PGF+ tip ECs (p = 0.0008) were significantly increased in NSCLC compared with normal lung tissues (**Figure S4C-S4E**). In another BRCA dataset with CD31+CD102+ ECs^33^, INSR+ tip ECs and PGF+ tip ECs were also confirmed. Both clusters were significantly elevated (INSR+: p = 0.018; PGF+: p = 0.029) in tumour tissues (**Figure S4F-S4H**). These external datasets confirmed the existence of these two tip EC phenotypes and verified the active angiogenesis in tumours (**Figure S4C-S4H**).

***INSR is a pan-cancer hallmark of tumour angiogenesis and a promising novel antiangiogenic target***

INSR+ tip ECs are the major component of tip TECs, with a mean fraction of 67.6% of all tip ECs (**Figure 3A**), disclosing the status of INSR+ tip ECs in tumour angiogenesis. We further examined the expression of INSR in tumour vasculature among diverse cancer types. IHC staining in multiple malignancies, including gastric cancer, ESCC and colorectal cancer (CRC), confirmed the particular enrichment of INSR in TECs, whereas NEC exhibited scarce INSR staining (**Figure S5B**). A further IHC staining in 20 types of cancer from the Human Protein Atlas (HPA) databases exhibited consistently robust enrichment of INSR in TECs, much higher than other components of the TME (**Figure S5C**). We further implemented mIF staining of CD31, INSRβ, and VEGFR2 to explore the expression of INSR in the TME. In 20 types of cancer tissues, INSRβ staining exhibited a uniform co-localization with CD31 staining, indicating the particular high expression of INSR in TECs. Besides, the INSRβ staining also co-localized with VEGFR2 staining, verifying its association with tumour angiogenesis (**Figure 3E**). The consistent expression of INSR in nearly 20 cancer types bolstered the credibility of INSR as a pan-cancer angiogenic marker.

The GO enrichment analyses of genes upregulated in INSR+ tip ECs revealed that pathways related with EC development, angiogenesis and insulin-like growth factor (IGF) binding were upregulated in INSR+ tip ECs (**Figure 4A-4B**). The upregulated IGF-binding pathways proposed the underlying pro-angiogenic functions of insulin and IGFs via INSR+ tip ECs. It’s reported that tumour-derived IGFs can promote angiogenesis, which was reported to be achieved via IGF1R^34, 35^. To confirm the pro-angiogenic effects of IGFs, we examined the correlationship between IGFs and classical EC markers (**Figure 4C**). Both *IGF1* and *IGF2* were significantly correlated with classical EC markers *PECAM1* and *VWF* in multiple cancers, verifying their pro-angiogenic effects (**Figure 4C**). This confirmed the angiogenic effects of IGFs, but what conflicts the literature was the relatively low expression levels of *IGF1R* and *IGF2R* in ECs (**Figure 4D-4E**). However, another high-affinity receptor of IGFs, insulin receptor^36^, was expressed exclusively in tumour ECs, and was identified as the marker for a newly identified tip EC cluster (**Figure 3A** and **Figure 4D-4E**).

Besides, we further explored the correlationship between the IGF receptors and classical EC markers (**Figure S6A**). The results showed that the correlations between *IGF1R* or *IGF2R* and classical EC markers *PECAM1* and *VWF* were weak in nearly all cancer types. In contrast, *INSR* exhibited a strong correlation with both *PECAM1* and *VWF* in multiple cancers (**Figure S6A**), indicating that the pro-angiogenic effects of IGFs might predominantly be achieved via insulin receptor, but not IGF1R and IGF2R. These results indicated that INSR plays an essential role in tumour angiogenesis via insulin/IGF1/IGF2-INSR axis.

Next, we tried to confirm the impacts of INSR on angiogenesis in both *in vitro* and *in vivo* assays. We first conducted *in vitro* Matrigel angiogenesis assays to evaluate the impact of INSR on the tube formation capacity of Human Umbilical Vein Endothelial Cells (HUVECs). Overexpression of *INSR* significantly enhanced the capacity of HUVECs to form tubes *in vitro* (**Figure 4F** and **Figure 4H**), while *INSR* knockdown impaired the formation of *in vitro* tubes (**Figure 4G** and **Figure 4I**), supporting the enhancement of angiogenic capacity via INSR.

Based on the strong effect of INSR in promoting angiogenesis, we asked whether INSR blockade could work with existing therapeutics to suppress the growth of tumours (**Figure 4J-4Q** and **Figure S6B-S6C**). Then we examined the therapeutic efficacy of BMS-754807, a small-molecule inhibitor of INSR and IGF-1R, in subcutaneous tumour models derived from two mouse cell lines. We observed that both LLC and CT26 tumours responded slightly to INSR inhibition alone, but the efficacy was inferior to the clinically commonly adopted strategy of chemotherapy plus VEGFA/VEGFR2 blockade. However, when combined with chemotherapy, INSR inhibition showed similar efficacy with VEGFR2 antibody and the efficacy reached its maximum when chemotherapy, VEGFR2 antibody and INSR inhibitor were administered simultaneously (**Figure 4J-4Q** and **Figure S6B-S6C**).

Using LLC orthotopic models followed by flow cytometry experiments to, we confirmed that both αVEGFR2 and INSR inhibitor could significantly reduce the abundance of ECs in orthotopic tumors, whether or not chemotherapy was combined (**Figure S7H** and **Figure 4O**). Besides, INSR inhibitor could significantly lower the expression level of VEGFR2 and INSR on ECs in orthotopic tumors, and the effects would be enhanced when αVEGFR2 were combined (**Figure 4P and 4Q**). Intriguingly, chemotherapy would increase the EC abundance in orthotopic tumours, and the effects could be reversed by INSR inhibitor (**Figure 4P and 4Q**).

In summary, we identified INSR as a landmark of tumour angiogenesis and proposed it to be a novel targetable agent. The *in vivo* and *in vitro* experiments demonstrated its vital status in tumour angiogenesis through its role in upregulating glucose uptake and metabolism.

***Mature venous ECs express MHC-II molecules and sensitize immunotherapy via MHC-II molecules***

Similar as other antigen-processing cells, ECs have also been reported to express genes involved in MHC-II-mediated antigen presentation and processing^21^. As the presence of MHC-II molecules is one of the major dominants of immunotherapy response, we then explored the expression level of MHC-II molecules in ECs. Intriguingly, mature ECs, including arterial, capillary and venous ECs, expressed a high level of MHC-II molecules; whereas naïve ECs, primarily immature ECs and sprouting tip ECs, expressed MHC-II molecules infrequently (**Figure S7A**- **S7B**). This suggests that the ECs might lose their antigen-presenting function during the angiogenic process, especially under the stimulation of various pro-angiogenic factors in the TME, such as VEGFA, PGF, ANGPT2 et al., which will activate the mature ECs and convert them into embryonic ECs. When comparing all EC clusters, we discovered that mature venous ECs expressed the highest level of MHC-II molecules (**Figure S7A**- **S7B**), and this high level of MHC-II molecules was present on nearly all venous EC subclusters (**Figure S2C**). According to KEGG enrichment analyses, antigen processing and presentation pathways were enriched in venous ECs (**Figure S2G**). Besides, the activated venous cluster (H8) exhibited a modest downregulation of MHC-II molecules (**Figure S2C**), further validating our hypothesis that the angiogenic factors in the TME will suppress the capacity of mature venous ECs to process neoantigen, thereby forming an immune-suppression TME.

The roles of ECs in tumour immunobiology and immunotherapy response remain largely unknown, thus we then analysed the relationship between EC composition and immunotherapy response. PICC (ClinicalTrials.gov: NCT03926338) is a neoadjuvant platform trial investigating the combined effects of Toripalimab and celecoxib in mismatch repair-proficient and microsatellite-stable (dMMR/MSI-H) colorectal cancer (CRC)^14^. We extracted ECs from the scRNA-seq data of dMMR/MSI-H CRC in the PICC trial for further investigation (**Figure S7C- S7E**). Major EC phenotypes were identified (**Figure S7C**). In this cohort, we observed remarkably enriched mature venous ECs in the tumours with complete immune checkpoint blocker (ICB) response, while tip ECs and immature ECs were enriched in the non-response group (**Figure S7D**). Further supporting these results, we observed a significant upregulation of multiple MHC-II molecules in ECs in response tumours (vs non-response; all p < 10e-16) (**Figure S7E**).

To increase the reliability, we extended this analysis to another independent cohort (**Figure S7F-S7H**). TRACERx (ClinicalTrials.gov: NCT03226886) is a trial examining the efficiency of ICB (Nivolumab or Ipilimumab) on advanced clear cell renal cell carcinoma^15^. ECs were extracted and cluttered to identify major EC lineage (**Figure S7F**). In this cohort, we also observed remarkably enriched mature venous ECs in the tumours with complete immune checkpoint blocker (ICB) response, and enriched tip ECs in the non-response group (**Figure S7G**). For normal kidney, urinary collecting duct ECs were enriched (**Figure S7G**). Further analyses validated the significant upregulation of multiple MHC-II molecules in ECs in response tumours (vs non-response; all p < 10e-16) (**Figure S7H**). These two ICB-treatment cohorts independently supported that mature venous ECs expressing high levels of MHC-II molecules are associated with better response to ICB treatments. Oppositely, the enrichment of immature components, which is the highlighted characteristic of tumour vessels as stated above, is related to the resistance of ICB treatments.

We then asked whether the levels of MHC-II+ venous ECs were related with ICB response. Using ICB-treatment cohorts with transcriptome data obtained from the Tumor Immune Dysfunction and Exclusion database (TIDE), we estimated the level of MHC-II+ venous ECs using ssGSVA method and investigated its relationship with ICB response^16^. There ICB cohorts were included and analysed independently, including the Gide2019 melanoma cohort (Nivolumab or Pembrolizumab)^17^, Riaz2017 melanoma cohort (Nivolumab)^18^, and Prat2017 multiple cancer cohort (Pembrolizumab)^19^. Notably, in the Gide2019 melanoma cohort profiled prior to ICB therapy, we observed a prognostic advantage in patients with high levels of MHC-II+ venous ECs (overall survival [OS]: Hazard ratio [HR]: 0.27, p = 0.001; progression-free survival [PFS]: HR = 0.25, p = 0.0007) (**Figure S7I** and **Figure S7L**). Consistently, high levels of MHC-II+ venous ECs after ICB treatments were also associated with a prognosis superiority (OS: HR = 0.23, p = 0.04; PFS: HR = 0.24, p = 0.023) (**Figure S7J** and **Figure S7M**). In another cohort included multiple cancers treated with ICB, the pre-ICB high level of MHC-II+ venous ECs also predicted better PFS (HR = 0.35, p = 0.023) (**Figure S7K**). In all of the three cohorts, high level of MHC-II+ venous ECs was associated with advanced response to ICB treatments (response rate [high vs. low]: Gide2019: 58.1% vs. 10.0%; Riaz2017: 50.0% vs. 0%; Prat2017: 73.1% vs. 28.6%) (**Figure S7N**).

Our pan-cancer TEC atlas illustrated the presence of MHC-II molecules in mature venous ECs, pinpoints their noteworthy function as antigen-presenting cells, and links it with immunotherapy response. Our results might provide new interpretations to the mechanisms underlying the combination of antiangiogenic therapy and immunotherapy in clinical practice^37, 38^. Given the high plasticity of ECs, tip ECs and immature ECs would develop into mature venous and arterial ECs; conversely, mature ECs would also be triggered by the superfluous angiogenic factors in TME to be dedifferentiated, which disable the function of mature ECs. The accumulation of dedifferentiated ECs, which lose antigen-presentation ability, would lead to immunotherapy resistance. Antiangiogenic agents could counteract the aberrantly elevated angiogenic factors in TME, reverse the dedifferentiation process of mature ECs, and preserve the MHC-II+ venous ECs’ capacity to mediate neoantigens presentation. Upon antiangiogenic treatments, the accumulated MHC-II+ mature venous ECs will restore the anti-cancer immunity, re-activate cytotoxic cells, and re-sensitize immunotherapy.

In summary, by integrating pan-cancer TEC data, this study comprehensively profiled the tumour angiogenic landscape. By integrating the features of various ECs, we proposed a novel model of tumour angiogenesis process, reflecting the complexity of regulatory in this process. INSR+ tip ECs were identified as the major angiogenic entity in neo-angiogenesis. In the angiogenic process, the upregulation of *INSR,* which enhances the angiogenic potency via IGF1/IGF2/insulin-INSR axis. This pan-cancer TEC atlas further linked MHC-II+ venous ECs with immunotherapy response.

**Refernece**

1. Zheng GX, Terry JM, Belgrader P, Ryvkin P, Bent ZW, Wilson R, Ziraldo SB, Wheeler TD, McDermott GP, Zhu J, Gregory MT, Shuga J, Montesclaros L, Underwood JG, Masquelier DA, Nishimura SY, Schnall-Levin M, Wyatt PW, Hindson CM, Bharadwaj R, Wong A, Ness KD, Beppu LW, Deeg HJ, McFarland C, Loeb KR, Valente WJ, Ericson NG, Stevens EA, Radich JP, Mikkelsen TS, Hindson BJ and Bielas JH. Massively parallel digital transcriptional profiling of single cells. *Nat Commun*. 2017;8:14049.

2. Hao Y, Hao S, Andersen-Nissen E, Mauck WM, 3rd, Zheng S, Butler A, Lee MJ, Wilk AJ, Darby C, Zager M, Hoffman P, Stoeckius M, Papalexi E, Mimitou EP, Jain J, Srivastava A, Stuart T, Fleming LM, Yeung B, Rogers AJ, McElrath JM, Blish CA, Gottardo R, Smibert P and Satija R. Integrated analysis of multimodal single-cell data. *Cell*. 2021;184:3573-3587 e29.

3. McGinnis CS, Murrow LM and Gartner ZJ. DoubletFinder: Doublet Detection in Single-Cell RNA Sequencing Data Using Artificial Nearest Neighbors. *Cell Syst*. 2019;8:329-337 e4.

4. Korsunsky I, Millard N, Fan J, Slowikowski K, Zhang F, Wei K, Baglaenko Y, Brenner M, Loh PR and Raychaudhuri S. Fast, sensitive and accurate integration of single-cell data with Harmony. *Nat Methods*. 2019;16:1289-1296.

5. Jin S, Guerrero-Juarez CF, Zhang L, Chang I, Ramos R, Kuan CH, Myung P, Plikus MV and Nie Q. Inference and analysis of cell-cell communication using CellChat. *Nat Commun*. 2021;12:1088.

6. Qiu X, Hill A, Packer J, Lin D, Ma YA and Trapnell C. Single-cell mRNA quantification and differential analysis with Census. *Nat Methods*. 2017;14:309-315.

7. Goldman MJ, Craft B, Hastie M, Repecka K, McDade F, Kamath A, Banerjee A, Luo Y, Rogers D, Brooks AN, Zhu J and Haussler D. Visualizing and interpreting cancer genomics data via the Xena platform. *Nat Biotechnol*. 2020;38:675-678.

8. Barrett T, Wilhite SE, Ledoux P, Evangelista C, Kim IF, Tomashevsky M, Marshall KA, Phillippy KH, Sherman PM, Holko M, Yefanov A, Lee H, Zhang N, Robertson CL, Serova N, Davis S and Soboleva A. NCBI GEO: archive for functional genomics data sets--update. *Nucleic Acids Res*. 2013;41:D991-5.

9. Athar A, Fullgrabe A, George N, Iqbal H, Huerta L, Ali A, Snow C, Fonseca NA, Petryszak R, Papatheodorou I, Sarkans U and Brazma A. ArrayExpress update - from bulk to single-cell expression data. *Nucleic Acids Res*. 2019;47:D711-D715.

10. Wu T, Hu E, Xu S, Chen M, Guo P, Dai Z, Feng T, Zhou L, Tang W, Zhan L, Fu X, Liu S, Bo X and Yu G. clusterProfiler 4.0: A universal enrichment tool for interpreting omics data. *Innovation (Camb)*. 2021;2:100141.

11. Hanzelmann S, Castelo R and Guinney J. GSVA: gene set variation analysis for microarray and RNA-seq data. *BMC Bioinformatics*. 2013;14:7.

12. Therneau, T. M. (2020). A Package for Survival Analysis in R. Available at <https://CRAN.R-project.org/package=survival>. Accessed on October 23, 2019.

13. Uhlen M, Fagerberg L, Hallstrom BM, Lindskog C, Oksvold P, Mardinoglu A, Sivertsson A, Kampf C, Sjostedt E, Asplund A, Olsson I, Edlund K, Lundberg E, Navani S, Szigyarto CA, Odeberg J, Djureinovic D, Takanen JO, Hober S, Alm T, Edqvist PH, Berling H, Tegel H, Mulder J, Rockberg J, Nilsson P, Schwenk JM, Hamsten M, von Feilitzen K, Forsberg M, Persson L, Johansson F, Zwahlen M, von Heijne G, Nielsen J and Ponten F. Proteomics. Tissue-based map of the human proteome. *Science*. 2015;347:1260419.

14. Li J, Wu C, Hu H, Qin G, Wu X, Bai F, Zhang J, Cai Y, Huang Y, Wang C, Yang J, Luan Y, Jiang Z, Ling J, Wu Z, Chen Y, Xie Z and Deng Y. Remodeling of the immune and stromal cell compartment by PD-1 blockade in mismatch repair-deficient colorectal cancer. *Cancer Cell*. 2023.

15. Krishna C, DiNatale RG, Kuo F, Srivastava RM, Vuong L, Chowell D, Gupta S, Vanderbilt C, Purohit TA, Liu M, Kansler E, Nixon BG, Chen YB, Makarov V, Blum KA, Attalla K, Weng S, Salmans ML, Golkaram M, Liu L, Zhang S, Vijayaraghavan R, Pawlowski T, Reuter V, Carlo MI, Voss MH, Coleman J, Russo P, Motzer RJ, Li MO, Leslie CS, Chan TA and Hakimi AA. Single-cell sequencing links multiregional immune landscapes and tissue-resident T cells in ccRCC to tumor topology and therapy efficacy. *Cancer Cell*. 2021;39:662-677 e6.

16. Fu J, Li K, Zhang W, Wan C, Zhang J, Jiang P and Liu XS. Large-scale public data reuse to model immunotherapy response and resistance. *Genome Med*. 2020;12:21.

17. Gide TN, Quek C, Menzies AM, Tasker AT, Shang P, Holst J, Madore J, Lim SY, Velickovic R, Wongchenko M, Yan Y, Lo S, Carlino MS, Guminski A, Saw RPM, Pang A, McGuire HM, Palendira U, Thompson JF, Rizos H, Silva IPD, Batten M, Scolyer RA, Long GV and Wilmott JS. Distinct Immune Cell Populations Define Response to Anti-PD-1 Monotherapy and Anti-PD-1/Anti-CTLA-4 Combined Therapy. *Cancer Cell*. 2019;35:238-255 e6.

18. Riaz N, Havel JJ, Makarov V, Desrichard A, Urba WJ, Sims JS, Hodi FS, Martin-Algarra S, Mandal R, Sharfman WH, Bhatia S, Hwu WJ, Gajewski TF, Slingluff CL, Jr., Chowell D, Kendall SM, Chang H, Shah R, Kuo F, Morris LGT, Sidhom JW, Schneck JP, Horak CE, Weinhold N and Chan TA. Tumor and Microenvironment Evolution during Immunotherapy with Nivolumab. *Cell*. 2017;171:934-949 e16.

19. Prat A, Navarro A, Pare L, Reguart N, Galvan P, Pascual T, Martinez A, Nuciforo P, Comerma L, Alos L, Pardo N, Cedres S, Fan C, Parker JS, Gaba L, Victoria I, Vinolas N, Vivancos A, Arance A and Felip E. Immune-Related Gene Expression Profiling After PD-1 Blockade in Non-Small Cell Lung Carcinoma, Head and Neck Squamous Cell Carcinoma, and Melanoma. *Cancer Res*. 2017;77:3540-3550.

20. Zhu L, Yu X, Wang L, Liu J, Qu Z, Zhang H, Li L, Chen J and Zhou Q. Angiogenesis and immune checkpoint dual blockade in combination with radiotherapy for treatment of solid cancers: opportunities and challenges. *Oncogenesis*. 2021;10:47.

21. Goveia J, Rohlenova K, Taverna F, Treps L, Conradi LC, Pircher A, Geldhof V, de Rooij L, Kalucka J, Sokol L, Garcia-Caballero M, Zheng Y, Qian J, Teuwen LA, Khan S, Boeckx B, Wauters E, Decaluwe H, De Leyn P, Vansteenkiste J, Weynand B, Sagaert X, Verbeken E, Wolthuis A, Topal B, Everaerts W, Bohnenberger H, Emmert A, Panovska D, De Smet F, Staal FJT, McLaughlin RJ, Impens F, Lagani V, Vinckier S, Mazzone M, Schoonjans L, Dewerchin M, Eelen G, Karakach TK, Yang H, Wang J, Bolund L, Lin L, Thienpont B, Li X, Lambrechts D, Luo Y and Carmeliet P. An Integrated Gene Expression Landscape Profiling Approach to Identify Lung Tumor Endothelial Cell Heterogeneity and Angiogenic Candidates. *Cancer Cell*. 2020;37:21-36 e13.

22. Cazejust J, Bessoud B, Colignon N, Garcia-Alba C, Planche O and Menu Y. Hepatocellular carcinoma vascularization: from the most common to the lesser known arteries. *Diagn Interv Imaging*. 2014;95:27-36.

23. Feng M, Pan Y, Kong R and Shu S. Therapy of Primary Liver Cancer. *Innovation (Camb)*. 2020;1:100032.

24. Monk BJ, Minion LE and Coleman RL. Anti-angiogenic agents in ovarian cancer: past, present, and future. *Ann Oncol*. 2016;27 Suppl 1:i33-i39.

25. Tang H, Li G, Liu C, Huang D, Zhang X, Qiu Y and Liu Y. Diagnosis of lymph node metastasis in head and neck squamous cell carcinoma using deep learning. *Laryngoscope Investig Otolaryngol*. 2022;7:161-169.

26. Rohlenova K, Goveia J, Garcia-Caballero M, Subramanian A, Kalucka J, Treps L, Falkenberg KD, de Rooij L, Zheng Y, Lin L, Sokol L, Teuwen LA, Geldhof V, Taverna F, Pircher A, Conradi LC, Khan S, Stegen S, Panovska D, De Smet F, Staal FJT, McLaughlin RJ, Vinckier S, Van Bergen T, Ectors N, De Haes P, Wang J, Bolund L, Schoonjans L, Karakach TK, Yang H, Carmeliet G, Liu Y, Thienpont B, Dewerchin M, Eelen G, Li X, Luo Y and Carmeliet P. Single-Cell RNA Sequencing Maps Endothelial Metabolic Plasticity in Pathological Angiogenesis. *Cell Metab*. 2020;31:862-877 e14.

27. Schaaf MB, Garg AD and Agostinis P. Defining the role of the tumor vasculature in antitumor immunity and immunotherapy. *Cell Death Dis*. 2018;9:115.

28. Bhandari S, Larsen AK, McCourt P, Smedsrod B and Sorensen KK. The Scavenger Function of Liver Sinusoidal Endothelial Cells in Health and Disease. *Front Physiol*. 2021;12:757469.

29. Haeusler RA, McGraw TE and Accili D. Biochemical and cellular properties of insulin receptor signalling. *Nature Reviews Molecular Cell Biology*. 2018;19:31-44.

30. Kim N, Kim HK, Lee K, Hong Y, Cho JH, Choi JW, Lee JI, Suh YL, Ku BM, Eum HH, Choi S, Choi YL, Joung JG, Park WY, Jung HA, Sun JM, Lee SH, Ahn JS, Park K, Ahn MJ and Lee HO. Single-cell RNA sequencing demonstrates the molecular and cellular reprogramming of metastatic lung adenocarcinoma. *Nat Commun*. 2020;11:2285.

31. Zhang J, Lu T, Lu S, Ma S, Han D, Zhang K, Xu C, Liu S, Gan L, Wu X, Yang F, Wen W and Qin W. Single-cell analysis of multiple cancer types reveals differences in endothelial cells between tumors and normal tissues. *Comput Struct Biotechnol J*. 2023;21:665-676.

32. Aoki S, Inoue K, Klein S, Halvorsen S, Chen J, Matsui A, Nikmaneshi MR, Kitahara S, Hato T, Chen X, Kawakubo K, Nia HT, Chen I, Schanne DH, Mamessier E, Shigeta K, Kikuchi H, Ramjiawan RR, Schmidt TC, Iwasaki M, Yau T, Hong TS, Quaas A, Plum PS, Dima S, Popescu I, Bardeesy N, Munn LL, Borad MJ, Sassi S, Jain RK, Zhu AX and Duda DG. Placental growth factor promotes tumour desmoplasia and treatment resistance in intrahepatic cholangiocarcinoma. *Gut*. 2022;71:185-193.

33. Geldhof V, de Rooij L, Sokol L, Amersfoort J, De Schepper M, Rohlenova K, Hoste G, Vanderstichele A, Delsupehe AM, Isnaldi E, Dai N, Taverna F, Khan S, Truong AK, Teuwen LA, Richard F, Treps L, Smeets A, Nevelsteen I, Weynand B, Vinckier S, Schoonjans L, Kalucka J, Desmedt C, Neven P, Mazzone M, Floris G, Punie K, Dewerchin M, Eelen G, Wildiers H, Li X, Luo Y and Carmeliet P. Single cell atlas identifies lipid-processing and immunomodulatory endothelial cells in healthy and malignant breast. *Nat Commun*. 2022;13:5511.

34. Zhang J, Liu M, Huang M, Chen M, Zhang D, Luo L, Ye G, Deng L, Peng Y, Wu X, Liu G, Ye W and Zhang D. Ginsenoside F1 promotes angiogenesis by activating the IGF-1/IGF1R pathway. *Pharmacol Res*. 2019;144:292-305.

35. Lin S, Zhang Q, Shao X, Zhang T, Xue C, Shi S, Zhao D and Lin Y. IGF-1 promotes angiogenesis in endothelial cells/adipose-derived stem cells co-culture system with activation of PI3K/Akt signal pathway. *Cell Prolif*. 2017;50.

36. Lodhia KA, Tienchaiananda P and Haluska P. Understanding the Key to Targeting the IGF Axis in Cancer: A Biomarker Assessment. *Front Oncol*. 2015;5:142.

37. Herbst RS, Arkenau HT, Santana-Davila R, Calvo E, Paz-Ares L, Cassier PA, Bendell J, Penel N, Krebs MG, Martin-Liberal J, Isambert N, Soriano A, Wermke M, Cultrera J, Gao L, Widau RC, Mi G, Jin J, Ferry D, Fuchs CS, Petrylak DP and Chau I. Ramucirumab plus pembrolizumab in patients with previously treated advanced non-small-cell lung cancer, gastro-oesophageal cancer, or urothelial carcinomas (JVDF): a multicohort, non-randomised, open-label, phase 1a/b trial. *Lancet Oncol*. 2019;20:1109-1123.

38. Khan KA and Kerbel RS. Improving immunotherapy outcomes with anti-angiogenic treatments and vice versa. *Nat Rev Clin Oncol*. 2018;15:310-324.

**Supplementary Figures**

**Figure S1. Landscape of the major components of the tumour microenvironment (TME) in pan-cancer data illustrated using scRNA-seq analysis.**

(A) Uniform manifold approximation and projection (UMAP) plots of pan-cancer samples with 14 TME clusters (left: nonimmune cells; right: immune cells). (B) Heatmap of the gene expression levels of the top-ranking marker genes in different TME clusters. In this and all further heatmaps depicting marker genes, the colours represent scaled gene expression. (C) UMAP plot of subclusters of T cells and NK cells. (D) UMAP plot of subclusters of myeloid cells. (E) UMAP plot of subclusters of B/plasma cells. (F) UMAP plot of subclusters of CAFs. (G) UMAP plot of subclusters of epithelial cells.

**Figure S2. Identifying endothelial cell (EC) phenotypes by scRNA-seq analysis.**

(A-D) Heatmaps of the expression levels of (A) arterial EC, (B) capillary EC, (C) venous EC, and (D) lymphatic endothelial cell (LEC) markers.

(E-H) Dot plot of Kyoto Encyclopedia of Genes and Genomes (KEGG) enrichment analyses of highly expressed genes of (E) arterial EC, (F) capillary EC, (G) venous EC, and (H) LEC markers. In each cluster, the top 5 upregulated pathways are illustrated.

**Figure S3. Comparisons of tumour endothelial cells (TECs) and normal endothelial cells (NECs).**

(A) Stacked bar plot comparing the relative abundance of major EC categories in TECs, NECs, and endothelial cells (ECs) in metastatic tumours (MetECs). (B) Stacked bar plot comparing the relative abundance of major EC categories in TECs, NECs, and MetECs in breast cancer (BRCA), hepatocellular carcinoma (HCC), lung adenocarcinoma (LUAD) and papillary thyroid tumour (PTC).

(C) Stacked bar plot comparing the relative abundance of arterial EC categories in arterial TECs and arterial NECs. (D) Stacked bar plot comparing the relative abundance of capillary EC categories in capillary TECs and capillary NECs. (E) Stacked bar plot comparing the relative abundance of immature EC categories in immature TECs and immature NECs. (F) Stacked bar plot comparing the relative abundance of lymphatic endothelial cell (LEC) categories in tumour LECs and normal LECs.

(G) Stacked bar plot comparing the relative abundance of venous EC phenotypes in venous TECs and venous NECs. (H) Box plot comparing the quantities of venous EC phenotypes in TECs and NECs (Wilcoxon test). The line and box represent the median and upper and lower quartiles, respectively.

(I) UMAP plot to show FCGR2B+ capillary ECs in liver NECs and HCC TECs. (J) UMAP plot to show FCGR2B+ capillary ECs in liver NECs and intrahepatic cholangiocarcinoma (ICC) TECs. (K) Bar plot to show the abundance of FCGR2B+ capillary ECs in TECs and NECs of various cancers and relevant normal tissues.

**Figure S4. Validation of angiogenic tip endothelial cells (ECs).**

(A) UMAP plot of tip ECs to show the expression of marker genes of the two tip EC phenotypes, including *INSR*, *FLT1*/*VEGFR1*, *PGF*, and *CXCR4*. (B) Violin plot comparing the expression of marker genes of the two tip EC phenotypes, including *INSR*, *FLT1*/*VEGFR1*, *PGF*, and *CXCR4*.

(C-E) Validation dataset 1: TECs and NECs in CD31+CD45- enriched cells from non-small cell lung cancer (NSCLC). (C) UMAP plot of tumour endothelial cells (TECs) and normal endothelial cells (NECs) in scRNA-seq data of NSCLC CD31+CD45- enriched cells (left: TECs and NECs combined; right: TECs and NECs separately). (D) Box plot comparing the quantities of tip ECs and capillary ECs in TECs and NECs in NSCLC CD31+CD45- enriched cells (Wilcoxon test). The line and box represent the median and upper and lower quartiles, respectively. (E) Box plot comparing the quantities of INSR+ tip ECs and PGF+ tip ECs in TECs and NECs in NSCLC CD31+CD45- enriched cells (Wilcoxon test). The line and box represent the median and upper and lower quartiles, respectively.

(F-H) Validation dataset 2: TECs and NECs in CD31+ CD102+ enriched cells from breast cancer (BRCA). (F) UMAP plot of TECs and NECs in scRNA-seq data of BRCA CD31+CD102+ enriched cells (left: TECs and NECs combined; right: TECs and NECs separately). (G) Box plot comparing the quantities of tip ECs and capillary ECs in TECs and NECs in BRCA CD31+CD102+ enriched cells (Wilcoxon test). The line and box represent the median and upper and lower quartiles, respectively. (H) Box plot comparing the quantities of INSR+ tip ECs and PGF+ tip ECs in TECs and NECs in BRCA CD31+CD102+ enriched cells (Wilcoxon test). The line and box represent the median and upper and lower quartiles, respectively.

**Figure S5. Expression levels of PGF and INSR.**

(A) Expression levels of *PGF* in endothelial cells and all other types of normal tissues. The expression levels were expressed in transcripts per million (TPM). All of the expression data were extracted from the HPA database.

(B) Immunohistochemical (IHC) staining of the β chain of *INSR* in primary tumour and normal tissues of colorectal cancer (CRC), oesophageal squamous cancer (ESCC), and gastric cancer (GC) (top: tumour tissues; bottom: normal tissues).

(C) IHC image of *INSR* staining in vessels of brain malignant glioma, breast duct carcinoma, cervical cancer, colon cancer, endometrium cancer, head and neck squamous carcinoma, kidney cancer, liver cancer, lung cancer, lymphoma involving the spleen, ovarian cancer, pancreas cancer, prostate cancer, skin melanoma, small intestinal cancer, stomach cancer, testis cancer, thyroid cancer, bladder cancer, and uterine cancer. All of the images were downloaded from the HPA database, and the image IDs are displayed on the images.

**Figure S6. INSR as a promising novel antiangiogenic target.**

(A) Correlation of *IGF1R*, *IGF2R* and *INSR* with the common endothelial cell (EC) markers of *CD31*/*PECAM1* and *VWF* in 32 cancer types of TCGA database. The horizontal axis is correlation coefficient and the vertical axis is the negative logarithm of p-value.

(B-E) *In vitro* Matrigel angiogenesis assays to investigate the tube-formation capacity of human umbilical vein endothelial cells (HUVECs). (B) *In vitro* tube-formation assay of HUVECs with or without *INSR* overexpression (oe*INSR*). (C) *In vitro* tube formation assay of HUVECs with or without INSR knockout with two independent siRNAs (sh*INSR*). (D) Western blot showing the level of the β chain of *INSR* in HUVECs with or without oe*INSR*. (E) Western blot showing the level of the β chain of *INSR* in HUVECs with or without sh*INSR*.

(F-G) Images of subcutaneous tumours derived from LLC (F) or CT26 (G) cells in different treatment groups.

(H) Dot plot showing the compositional changes of ECs by different interventions in LLC orthotopic models.

**Figure S7. Mature venous endothelial cells (ECs) express MHC-II molecules and sensitize immunotherapy via MHC-II molecules.**

(A) Uniform manifold approximation and projection (UMAP) plot of ECs to show the expression of common MHC-II molecules. (F) Violin plot comparing the expression of common MHC-II molecules in principle EC lineage.

(C-E) Analyses based on the single-cell transcriptome data involving ICB treatments in locally advanced dMMR colorectal cancer (CRC) from the PICC trial. (C) UMAP plot of ECs. (D) Enriched EC lineages in response (pCR) and non-response (non-pCR) groups. (E) Violin plot comparing the expression of common MHC-II molecules in pCR and non-pCR groups (Wilcoxon test).

(F-H) Analyses based on the single-cell transcriptome data involving ICB treatments in advanced clear cell renal cell carcinoma from the TRACERx trial. (F) UMAP plot of ECs. (G) Enriched EC lineages in pCR and non-pCR groups. (H) Violin plot comparing the expression of common MHC-II molecules in pCR and non-pCR groups (Wilcoxon test).

(I-N) Prognosis analyses based on the transcriptome data involving ICB treatments from the Tumor Immune Dysfunction and Exclusion (TIDE) database. (I) Overall survival (OS) and (L) progression-free survival (PFS) according to the levels of MHC-II+ venous ECs in Gide2019 melanoma cohort. (J) OS and (M) PFS according to the levels of MHC-II+ venous ECs in Riaz2017 melanoma cohort. (K) PFS according to the levels of MHC-II+ venous ECs in Prat2017 multiple primary cancer cohort. (N) Stack plot to comparing the response rate in the three cohort. Log-rank test was used to test the statistical significance in survival analyses.

**Supplementary Table Legend**

**Supplementary Table 1** The information of data set included in the pan-cancer single cell analysis.

**Supplementary Table 2** The information of samples included in the pan-cancer single cell analysis.


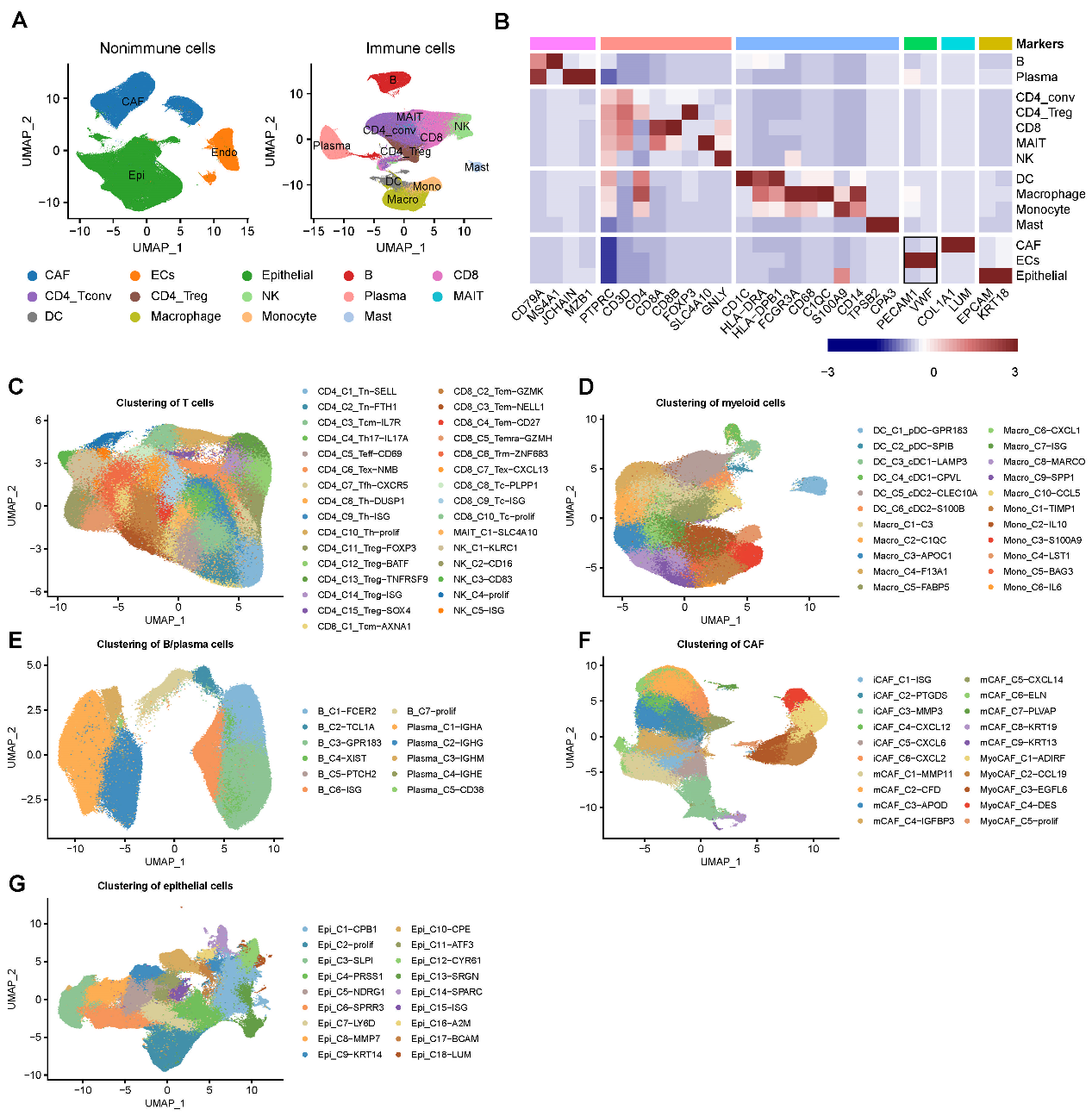


**Figure S1. Landscape of the major components of the tumour microenvironment (TME) in pan-cancer data illustrated using scRNA-seq analysis.**

(A) Uniform manifold approximation and projection (UMAP) plots of pan-cancer samples with 14 TME clusters (left: nonimmune cells; right: immune cells). (B) Heatmap of the gene expression levels of the top-ranking marker genes in different TME clusters. In this and all further heatmaps depicting marker genes, the colours represent scaled gene expression. (C) UMAP plot of subclusters of T cells and NK cells. (D) UMAP plot of subclusters of myeloid cells. (E) UMAP plot of subclusters of B/plasma cells. (F) UMAP plot of subclusters of CAFs. (G) UMAP plot of subclusters of epithelial cells.


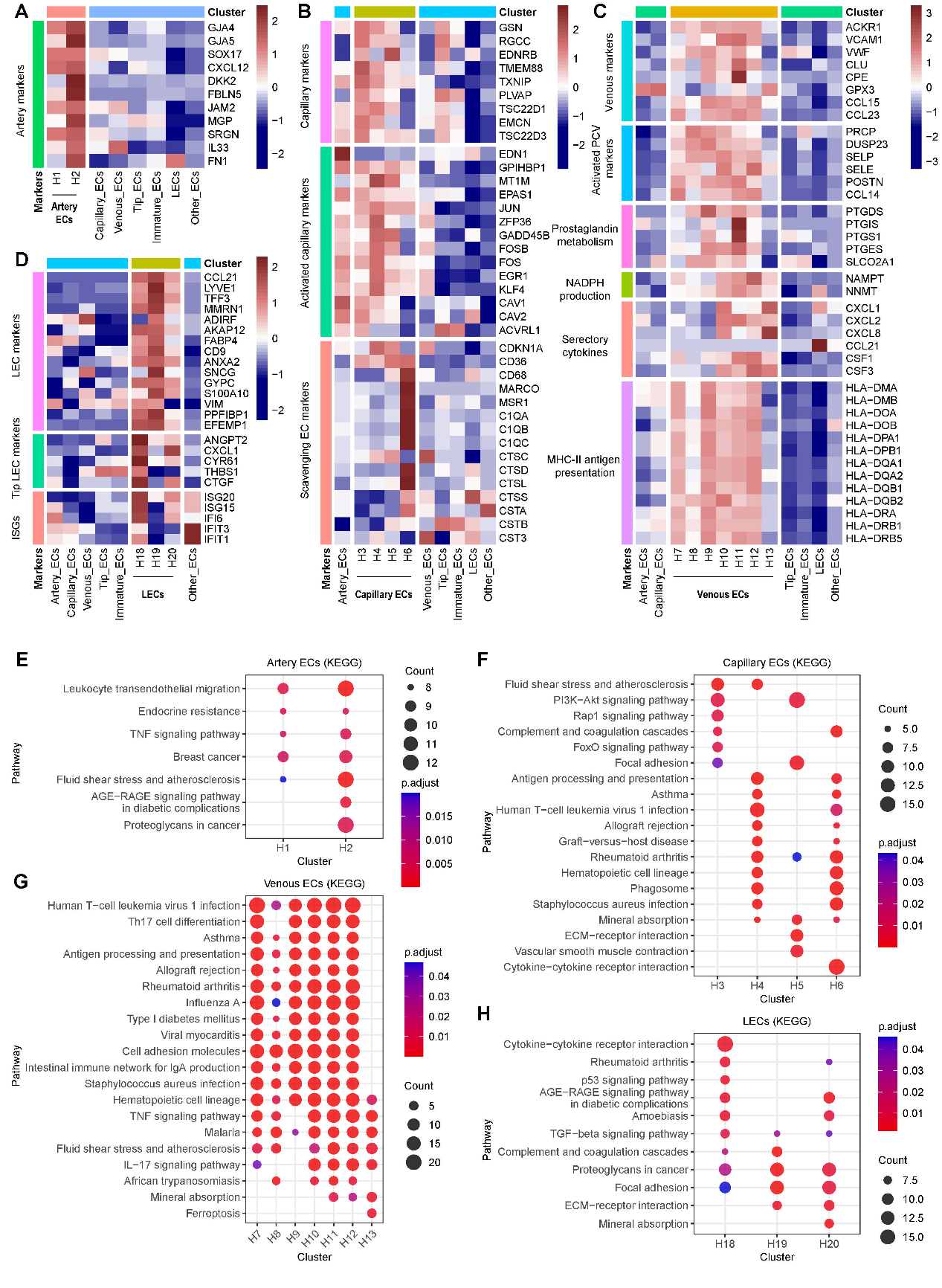


**Figure S2. Identifying endothelial cell (EC) phenotypes by scRNA-seq analysis.**

(A-D) Heatmaps of the expression levels of (A) arterial EC, (B) capillary EC, (C) venous EC, and (D) lymphatic endothelial cell (LEC) markers.

(E-H) Dot plot of Kyoto Encyclopedia of Genes and Genomes (KEGG) enrichment analyses of highly expressed genes of (E) arterial EC, (F) capillary EC, (G) venous EC, and (H) LEC markers. In each cluster, the top 5 upregulated pathways are illustrated.


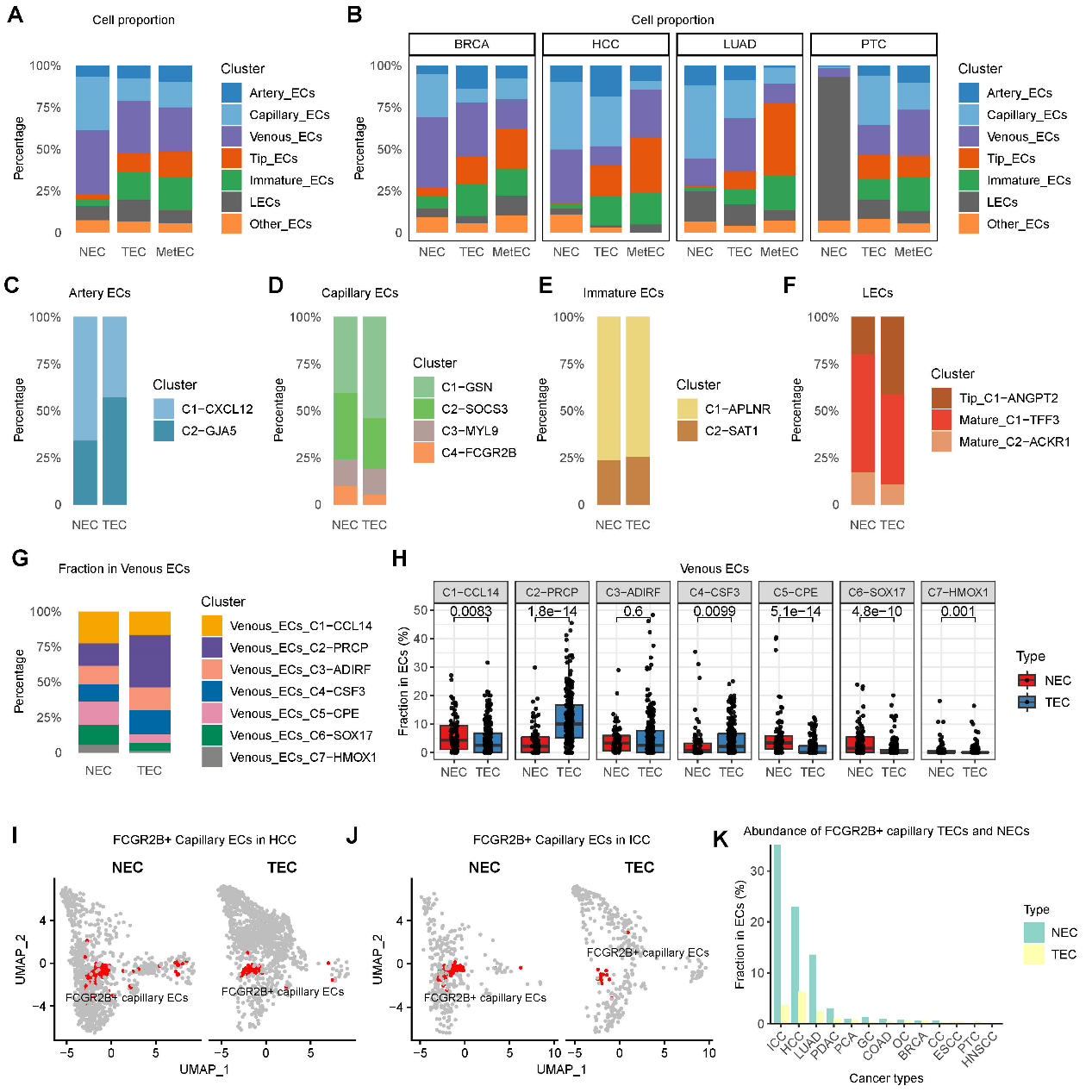


**Figure S3. Comparisons of** **tumour endothelial cells (TECs) and normal endothelial cells (NECs).**

(A) Stacked bar plot comparing the relative abundance of major EC categories in TECs, NECs, and endothelial cells (ECs) in metastatic tumours (MetECs). (B) Stacked bar plot comparing the relative abundance of major EC categories in TECs, NECs, and MetECs in breast cancer (BRCA), hepatocellular carcinoma (HCC), lung adenocarcinoma (LUAD) and papillary thyroid tumour (PTC).

(C) Stacked bar plot comparing the relative abundance of arterial EC categories in arterial TECs and arterial NECs. (D) Stacked bar plot comparing the relative abundance of capillary EC categories in capillary TECs and capillary NECs. (E) Stacked bar plot comparing the relative abundance of immature EC categories in immature TECs and immature NECs. (F) Stacked bar plot comparing the relative abundance of lymphatic endothelial cell (LEC) categories in tumour LECs and normal LECs.

(G) Stacked bar plot comparing the relative abundance of venous EC phenotypes in venous TECs and venous NECs. (H) Box plot comparing the quantities of venous EC phenotypes in TECs and NECs (Wilcoxon test). The line and box represent the median and upper and lower quartiles, respectively.

(I) UMAP plot to show FCGR2B+ capillary ECs in liver NECs and HCC TECs. (J) UMAP plot to show FCGR2B+ capillary ECs in liver NECs and intrahepatic cholangiocarcinoma (ICC) TECs. (K) Bar plot to show the abundance of FCGR2B+ capillary ECs in TECs and NECs of various cancers and relevant normal tissues.


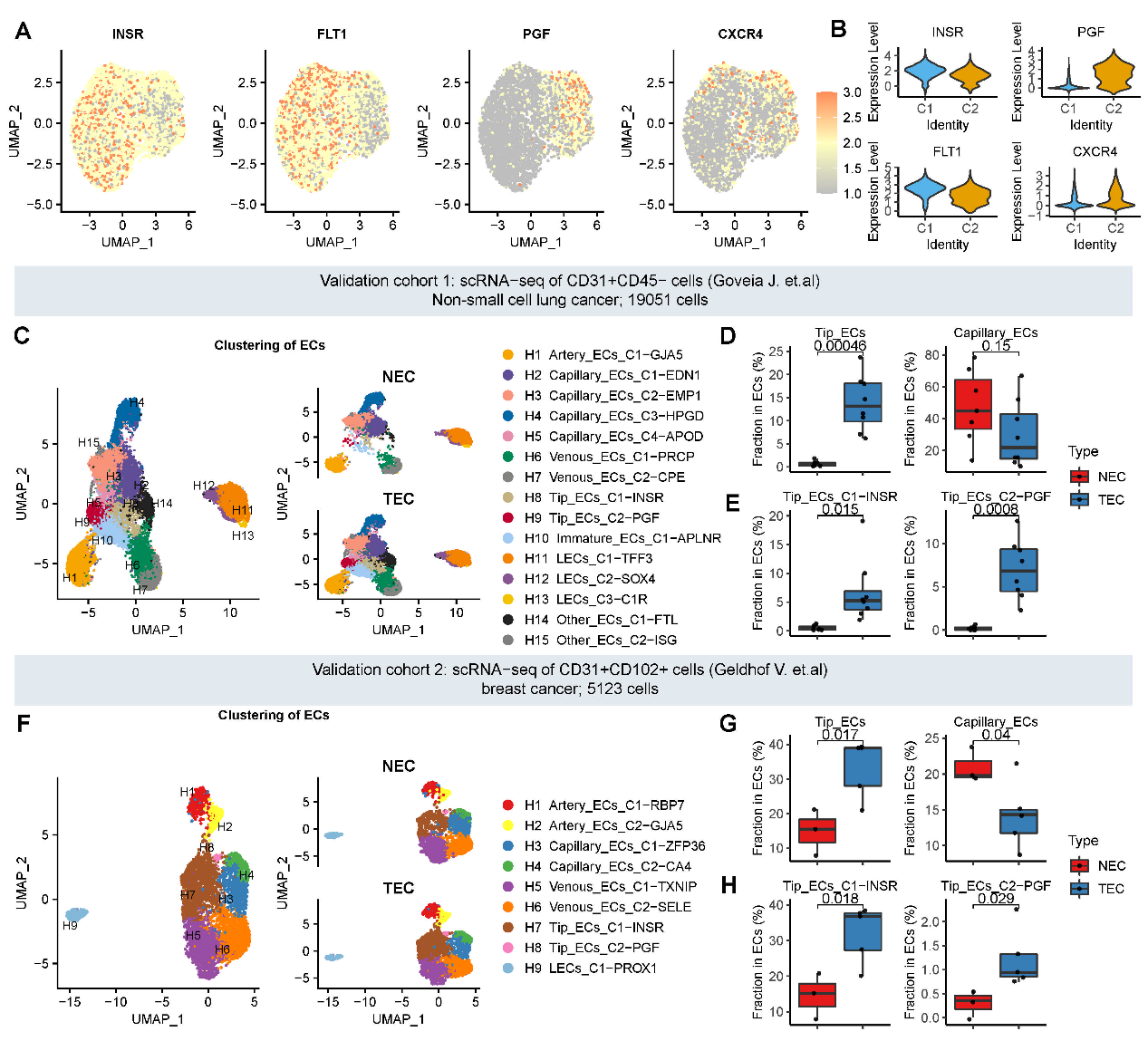


**Figure S4. Validation of angiogenic tip endothelial cells (ECs).**

(A) UMAP plot of tip ECs to show the expression of marker genes of the two tip EC phenotypes, including *INSR*, *FLT1*/*VEGFR1*, *PGF*, and *CXCR4*. (B) Violin plot comparing the expression of marker genes of the two tip EC phenotypes, including *INSR*, *FLT1*/*VEGFR1*, *PGF*, and *CXCR4*.

(C-E) Validation dataset 1: TECs and NECs in CD31+CD45- enriched cells from non-small cell lung cancer (NSCLC). (C) UMAP plot of tumour endothelial cells (TECs) and normal endothelial cells (NECs) in scRNA-seq data of NSCLC CD31+CD45- enriched cells (left: TECs and NECs combined; right: TECs and NECs separately). (D) Box plot comparing the quantities of tip ECs and capillary ECs in TECs and NECs in NSCLC CD31+CD45- enriched cells (Wilcoxon test). The line and box represent the median and upper and lower quartiles, respectively. (E) Box plot comparing the quantities of INSR+ tip ECs and PGF+ tip ECs in TECs and NECs in NSCLC CD31+CD45- enriched cells (Wilcoxon test). The line and box represent the median and upper and lower quartiles, respectively.

(F-H) Validation dataset 2: TECs and NECs in CD31+ CD102+ enriched cells from breast cancer (BRCA). (F) UMAP plot of TECs and NECs in scRNA-seq data of BRCA CD31+CD102+ enriched cells (left: TECs and NECs combined; right: TECs and NECs separately). (G) Box plot comparing the quantities of tip ECs and capillary ECs in TECs and NECs in BRCA CD31+CD102+ enriched cells (Wilcoxon test). The line and box represent the median and upper and lower quartiles, respectively. (H) Box plot comparing the quantities of INSR+ tip ECs and PGF+ tip ECs in TECs and NECs in BRCA CD31+CD102+ enriched cells (Wilcoxon test). The line and box represent the median and upper and lower quartiles, respectively.


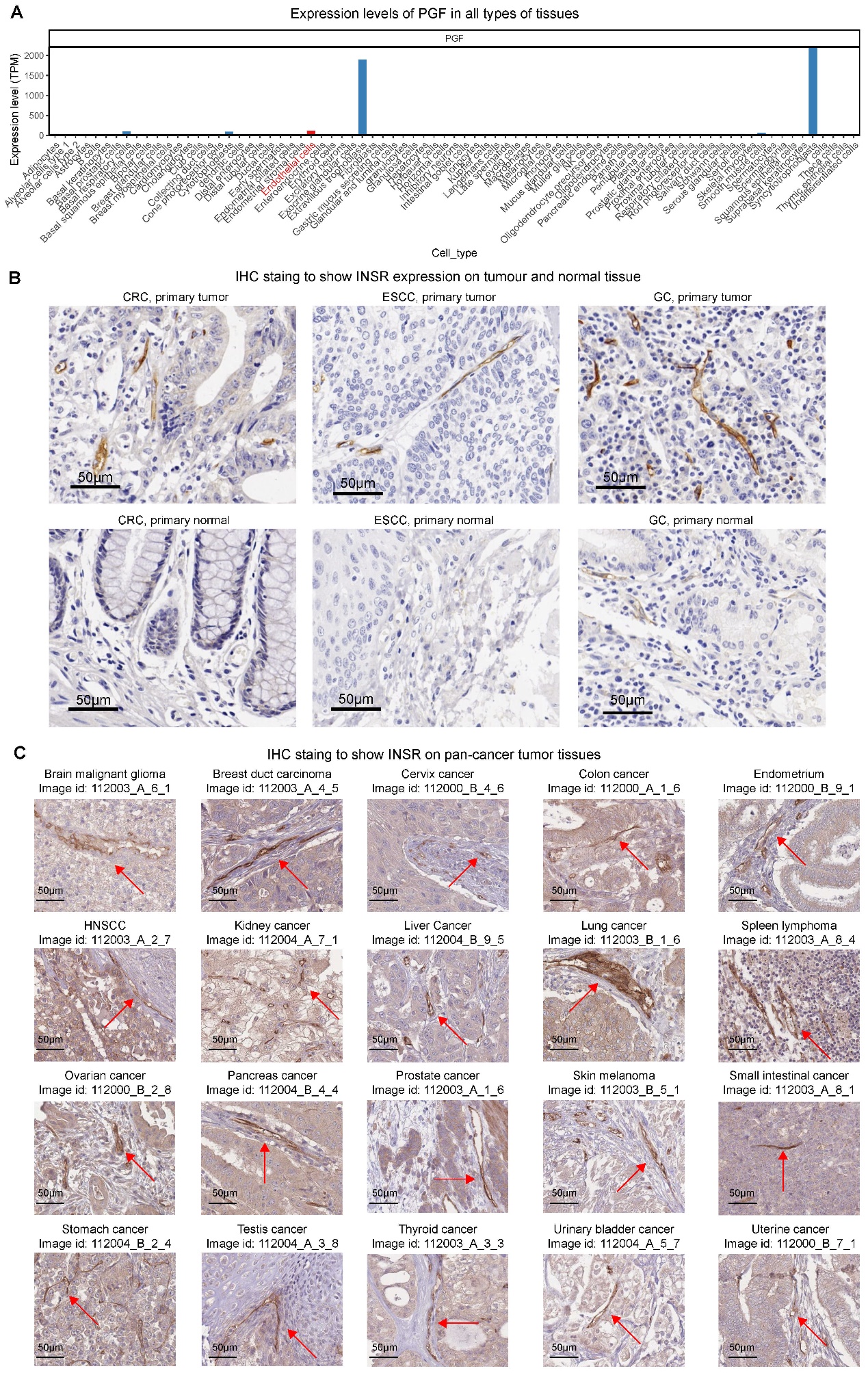


**Figure S5. Expression levels of PGF and INSR.**

(A) Expression levels of *PGF* in endothelial cells and all other types of normal tissues. The expression levels were expressed in transcripts per million (TPM). All of the expression data were extracted from the HPA database.

(B) Immunohistochemical (IHC) staining of the β chain of *INSR* in primary tumour and normal tissues of colorectal cancer (CRC), oesophageal squamous cancer (ESCC), and gastric cancer (GC) (top: tumour tissues; bottom: normal tissues).

(C) IHC image of *INSR* staining in vessels of brain malignant glioma, breast duct carcinoma, cervical cancer, colon cancer, endometrium cancer, head and neck squamous carcinoma, kidney cancer, liver cancer, lung cancer, lymphoma involving the spleen, ovarian cancer, pancreas cancer, prostate cancer, skin melanoma, small intestinal cancer, stomach cancer, testis cancer, thyroid cancer, bladder cancer, and uterine cancer. All of the images were downloaded from the HPA database, and the image IDs are displayed on the images.


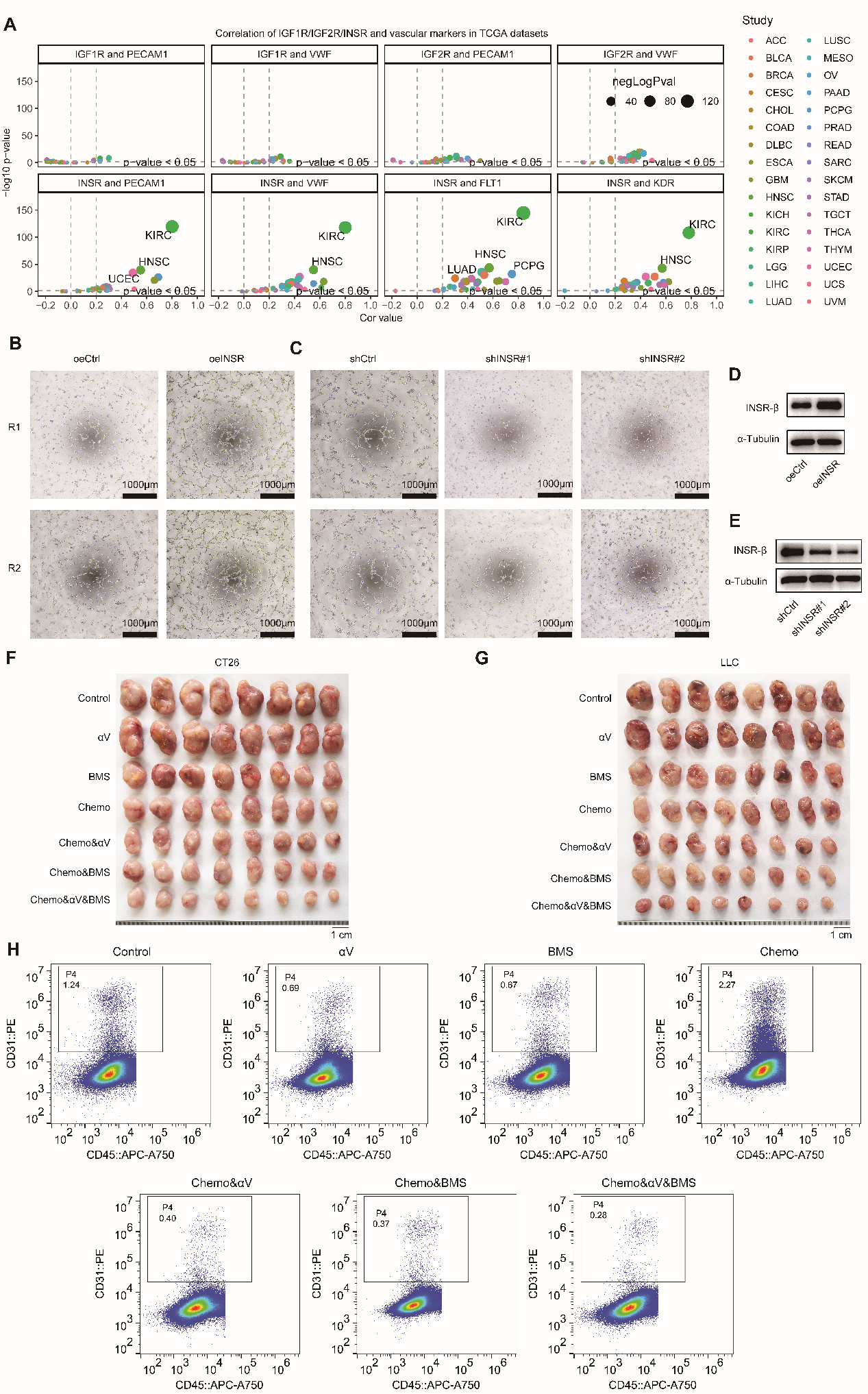


**Figure S6. INSR as a promising novel antiangiogenic target.**

(A) Correlation of *IGF1R*, *IGF2R* and *INSR* with the common endothelial cell (EC) markers of *CD31*/*PECAM1* and *VWF* in 32 cancer types of TCGA database. The horizontal axis is correlation coefficient and the vertical axis is the negative logarithm of p-value.

(B-E) *In vitro* Matrigel angiogenesis assays to investigate the tube-formation capacity of human umbilical vein endothelial cells (HUVECs). (B) *In vitro* tube-formation assay of HUVECs with or without *INSR* overexpression (oe*INSR*). (C) *In vitro* tube formation assay of HUVECs with or without INSR knockout with two independent siRNAs (sh*INSR*). (D) Western blot showing the level of the β chain of *INSR* in HUVECs with or without oe*INSR*. (E) Western blot showing the level of the β chain of *INSR* in HUVECs with or without sh*INSR*.

(F-G) Images of subcutaneous tumours derived from LLC (F) or CT26 (G) cells in different treatment groups.

(H) Dot plot showing the compositional changes of ECs by different interventions in LLC orthotopic models.


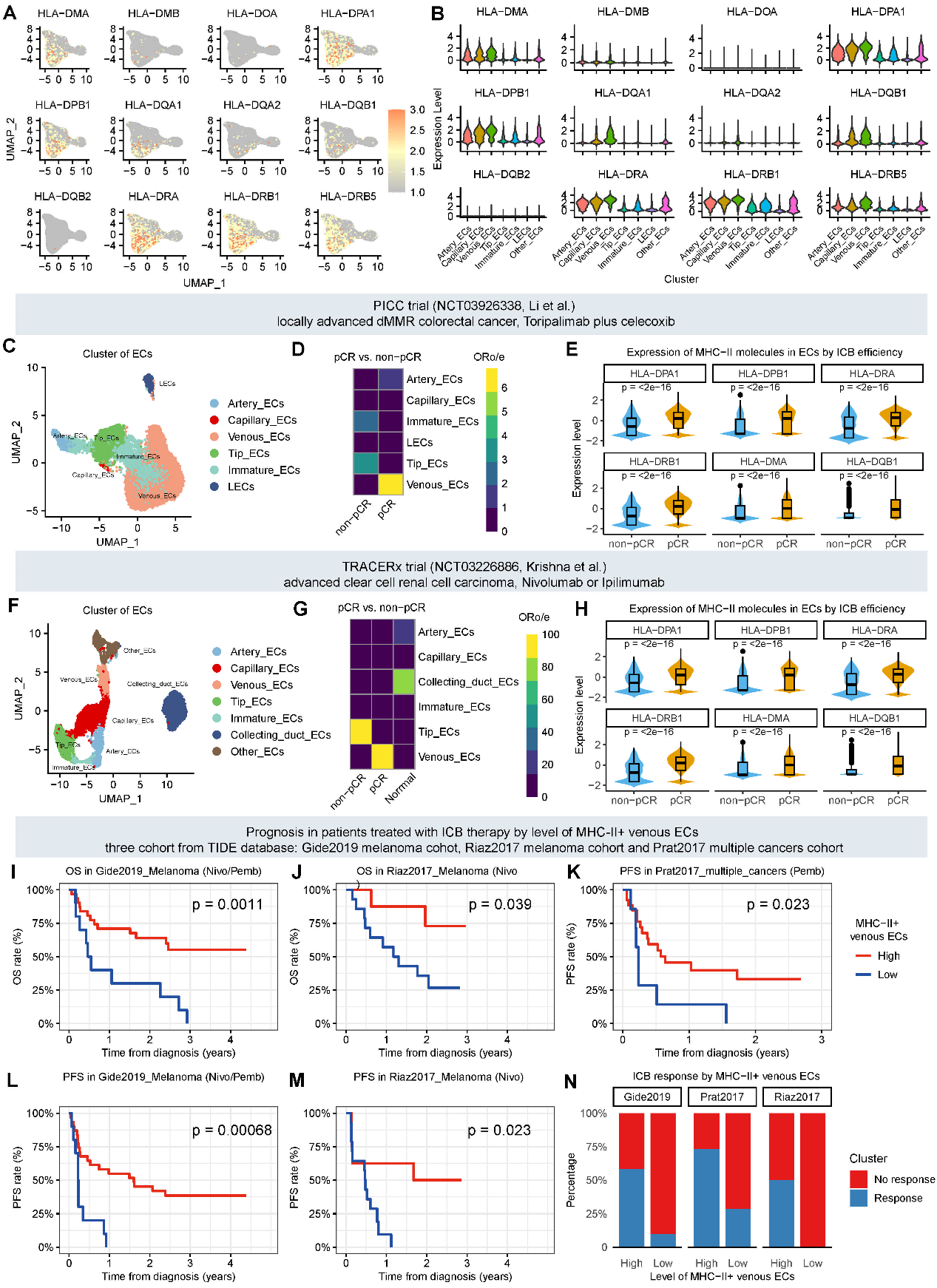


**Figure S7. Mature venous endothelial cells (ECs) express MHC-II molecules and sensitize immunotherapy via MHC-II molecules.**

(A) Uniform manifold approximation and projection (UMAP) plot of ECs to show the expression of common MHC-II molecules. (F) Violin plot comparing the expression of common MHC-II molecules in principle EC lineage.

(C-E) Analyses based on the single-cell transcriptome data involving ICB treatments in locally advanced dMMR colorectal cancer (CRC) from the PICC trial. (C) UMAP plot of ECs. (D) Enriched EC lineages in response (pCR) and non-response (non-pCR) groups. (E) Violin plot comparing the expression of common MHC-II molecules in pCR and non-pCR groups (Wilcoxon test).

(F-H) Analyses based on the single-cell transcriptome data involving ICB treatments in advanced clear cell renal cell carcinoma from the TRACERx trial. (F) UMAP plot of ECs. (G) Enriched EC lineages in pCR and non-pCR groups. (H) Violin plot comparing the expression of common MHC-II molecules in pCR and non-pCR groups (Wilcoxon test).

(I-N) Prognosis analyses based on the transcriptome data involving ICB treatments from the Tumor Immune Dysfunction and Exclusion (TIDE) database. (I) Overall survival (OS) and (L) progression-free survival (PFS) according to the levels of MHC-II+ venous ECs in Gide2019 melanoma cohort. (J) OS and (M) PFS according to the levels of MHC-II+ venous ECs in Riaz2017 melanoma cohort. (K) PFS according to the levels of MHC-II+ venous ECs in Prat2017 multiple primary cancer cohort. (N) Stack plot to comparing the response rate in the three cohort. Log-rank test was used to test the statistical significance in survival analyses.
